# Context and working memory capacity affect the processing of written irony in chinese: an eye-tracking study

**DOI:** 10.1007/s00426-026-02258-w

**Published:** 2026-03-31

**Authors:** Lijuan Zou, Zhijun Zhang, Xiaoyu Cheng, Yue Ma, Jukka Hyönä, Shouxin Li

**Affiliations:** 1 Faculty of Psychology, Shandong Normal University, Jinan, Shandong Province China; 2 Shandong Provincial Key Laboratory of Brain Science and Mental Health, Jinan, China; 3 Jinan Vocational College of nursing, Jinan, China; 4 Department of Psychology and Speech-Language Pathology, University of Turku, Turku, Finland

## Abstract

Written irony is defined as the use of words to express the opposite to the literal meaning. Comprehending ironic praise is more difficult than that of ironic criticism. However, whether and how language context and individuals’ working memory capacity (WMC) affect irony comprehension in Chinese whose script is distinct from alphabetic languages are currently unclear and warrant further research. In this study, participants read ironic criticism and ironic praise sentences while their eye movements were recorded. The results showed that the processing of ironic praise needed longer first reading time, regression path time, and total reading time as compared to the ironic criticism. However, no significant difference was found between literal criticism and literal praise. In addition, when the inconsistency between context and the ironic utterance was weak, first-pass and rereading time for ironic praise were significantly longer than those for ironic criticism. However, there was no difference between ironic praise and criticism in the strong context inconsistency condition. Furthermore, participants with low WMC showed significantly longer total reading time for ironic praise than participants with high WMC. Moreover, they read the prior context of ironic praise with longer regression path reading time and rereading time than that of ironic criticism. Taken together, these results suggest that ironic praise is more difficult to read and require more cognitive resources relative to ironic criticism in Chinese, which was similar with findings in Western cultures and scripts. More importantly, prior context and WMC modulated differently the influence on irony comprehension. Specifically, the weak context inconsistency made it harder to understand the ironic praise than ironic criticism, lasting from the early to the late processing stages. However, the influence of WMC mainly occurred in the late integration stage of irony comprehension. This study makes two major theoretical contributions. First, it extends key findings on irony processing—the greater difficulty of understanding ironic praise and the role of context and working memory—to Chinese, a distinct language and cultural system, by confirming that these cognitive mechanisms are universal. Second, it clarifies how these factors operate at different stages of processing and suggests that features of a writing system (such as logographic script) can influence the timing of high-level language comprehension.

**Supplementary Information:**

The online version contains supplementary material available at 10.1007/s00426-026-02258-w.

## Introduction

Written irony is defined as the use of words to express the opposite to the literal meaning (Attardo, 2000). It is commonly used in both Western and Eastern cultures. Irony can convey humor, criticism, or mockery, and is frequently used in daily language communication (Gibbs, 2000; Whalen et al., 2009). The Standard Pragmatic Model (SPM) (Grice, 1975) suggests that all forms of irony are processed via a three-step process. First, the literal meaning of the utterance is processed. Second, a possible discrepancy is detected between the literal meaning and the context in which it is presented. Finally, the reader makes an alternative interpretation and comprehends that the statement is irony. According to the SPM, irony comprehension should be more difficult and need more time than literal statement. Conversely, the Direct Access View (Gibbs, 1994) assumed that contextual information interacts with lexical processes very early, similar underlying mechanisms were involved in the initial processing of both literal and figurative languages. According to the direct access view, understanding irony does not necessarily require special cognitive processes beyond those used to comprehend literal language. However, it remains unclear whether irony and literal language reading in Chinese are similar or exhibit significant differences.

Irony mainly consists of two types: ironic criticism and ironic praise (Hancock et al., 2000; Huang & Wang, 2013; Zhang & Zhang, 2006). Ironic criticism (e.g., “You’re a great friend” following betrayal) refers to using positive language to express a negative intention. On the other hand, ironic praise (e.g., “What a terrible performance” after excellence) refers to using negative language to express a positive intention. Previous studies have revealed that the cognitive processing difficulty in comprehending ironic praise is greater than that of ironic criticism (Colston, 2000; Hancock et al., 2000; Pexman & Zvaigzne, 2004; Pexman & Glenwright, 2007). Researchers have found that children at 6–10 years of age can judge the intention of ironic criticism more accurately than that of ironic praise (Pexman & Glenwright, 2007). Moreover, ironic praise elicits a larger N400 component than literal language, while no significant difference was found between ironic criticism and literal language, indicating that ironic praise elicits a stronger semantic conflict than ironic criticism (Baptista et al., 2018; Caillies et al., 2019). Various proposals have been put forth to explain the processing difference between ironic criticism and praise. Gibbs’ (1986) normative context theory posits that irony comprehension relies on the violation of shared expectations: ironic criticism subverts positive norms (e.g., politeness), whereas ironic praise violates negative defaults, demanding greater inferential effort. Frequency-based expectation effects (Regel et al., 2010) further explain this disparity, as ironic criticism is more conventionalized in daily communication, leading to faster processing. The Pretense Theory (Clark & Gerrig, 1984) states that ironic criticism tends to imply norms and expectations about politeness and speaking nicely. Therefore, ironic criticism may not require explicit antecedent statements to be understood; instead, it only needs to echo or imply positive expectations people tend to hold of their own life and of the world in general. In contrast, the literal meaning of ironic praise is negative in its tone and has less predication towards the positive expectations or behavioral norms people implicitly hold. Thus, the comprehension of ironic praise requires more explicit prior context information (Hancock et al., 2000), and if the prior context information is not clear enough, its understanding will be significantly impeded (Gibbs, 1994; Utsumi, 2000). All frameworks highlight context as a critical moderator—especially for ironic praise, which demands explicit contextual support to override its negative literal meaning (Hancock et al., 2000) and to compensate for its lower frequency (Regel et al., 2010) or weaker normative violation (Gibbs, 1986).

A large number of studies from alphabetic languages have confirmed that context is one factor in the multidimensional interplay including linguistic cues, individual cognitive cues, and other limiting factors (Read & Miller, 1998) to effectively support the cognitive processing of irony (Colston, 2002; Colston & O’Brien, 2000; Gerrig & Goldvarg, 2000; Ivanko & Pexman, 2003; Katz, 2005; Katz & Ferretti, 2001; Pexman, 2008; Read & Miller, 1998; Rivière & Champagne-Lavau, 2020). The degree of consistency between the context and the ironic statement indicates the clarity of contextual information. Clear contextual information can speed up the individual’s comprehension of the discourse (Gibbs, 1994; Utsumi, 2000). Researchers have found that when there is strong inconsistency between the context and the literal meaning of the target statement, the context information points to an ironic interpretation and can thus facilitate the processing of irony. Conversely, when there is only weak inconsistency between the context and the literal meaning of the target statement, the context information does not clearly point to an ironic interpretation, which may impede irony processing (Rivière et al., 2018). As ironic praise is more difficult to comprehend than ironic criticism, contextual cues may exert a stronger effect on processing ironic praise than ironic criticism. Therefore, it was expected in the present study that when the context inconsistency is weak, the comprehension of ironic praise will be impeded, as indexed by longer processing time needed to comprehend ironic praise than ironic criticism. Conversely, when the inconsistency is strong, there will be no significant processing difference between ironic praise and ironic criticism.

Working memory capacity (WMC) is an important reader-related factor influencing irony processing (Read & Miller, 1998). Readers with high WMC can better maintain multiple potential interpretations in their mind and smoothly integrate pragmatic, semantic, and syntactic information in order to build a coherent internal representation of the text (Just & Carpenter, 1992). High WMC also means that more cognitive resources can be allocated to various reading requirements, such as basic visual information processing and integration of context and other top-down information including readers’ prior knowledge of the text’s contents (Kaakinen et al., 2003). As regards written irony comprehension, it was found that high WMC is significantly related to increased immediate rereading time of the ironic utterances (Kaakinen et al., 2014; Olkoniemi et al., 2016, 2019). Individuals with high WMC produced more immediate rereading during the first-pass reading of ironic statements, while those with low WMC responded to irony with some delay, as indexed by increased regression time spent during the reprocessing of ironic statements. The probability of rereading reflects an individual’s reanalysis of uncertain or difficult-to-understand information during reading. These findings suggest that high WMC readers are able to start processing the intended meaning early, but readers with low WMC may use compensatory strategies, such as looking back in text to make sense of ironic statements. However, it is not clear how working memory capacity may affect the processing of ironic praise and ironic criticism. As reviewed above, under weak context conditions, the processing of ironic praise is significantly harder than that of ironic criticism (Gibbs, 1994; Rivière et al., 2018; Utsumi, 2000). Thus, we reasoned in the present study that under weak context conditions the influence of WMC on the comprehension of ironic praise and ironic criticism may become more salient.

In summary, both context-related and reader-related factors such as working memory capacity affect irony comprehension (Olkoniemi & Kaakinen, 2021; Voyer et al., 2014). Notably, most research on irony comprehension has been conducted in North America and Western European countries. Researchers have proposed that there are significant cross-cultural differences in cognitive processing, from lower to higher level aspects of cognition (Nisbett et al., 2001). Thus, it should not be taken at face value that findings and conclusions based on studies conducted in Western countries generalize to other cultures and languages. The monosyllabic nature of Chinese characters and the abundance of homophones make Chinese irony more context-dependent (Jia et al., 2019), requiring stronger reliance on contextual information for interpretation. Thus, it is important to test whether script differences (Li et al., 2022) modulate the timeline of irony effects, as irony comprehension in logographic script has been studied only to a limited extent (Jia et al., 2019; Shi & Li, 2022). The present study contributes to this line of research by using eye-tracking to tap into the timeline of processing ironic praise and criticism during written language comprehension in Chinese. The present study explores how context and reader-related factors affect the cognitive processing of ironic criticism and ironic praise by conceptualizing irony processing in the context of the reading comprehension framework (Snow, 2002) including factors related to the reader (working memory capacity), text (weakly vs. strongly supporting context for ironic praise and ironic criticism), task (reading to answer questions) and sociocultural context (Chinese).

The main research question of the present study was whether and how context and working memory capacity affect the cognitive processing of ironic criticism and ironic praise in Chinese. Firstly, we tested whether Chinese irony processing had similar or dissimilar mechanisms with literal language and how the praise and criticism irony were processed differently. The expression type (literal vs. ironic) and evaluation type (praise vs. criticism) were manipulated in Experiment 1 to investigate the above-mentioned question. In Experiment 2, the degree of context consistency (weak vs. strong) was manipulated between the prior context and the ironic statement to test how varying degrees of contextual support modulate the cognitive demand inherent in irony comprehension. Strong inconsistency represents prototypical irony with explicit context-statement clashes (e.g., praising a mess as “clean” violates politeness norms), while weak inconsistency reflects non-prototypical use of irony requiring a pragmatic inference to detect normative subversion. In Experiment 3, we aimed to test how high and low working memory capacity influences the cognitive processing of ironic criticism and ironic praise. Eye-tracking technology, which is well suited to study text comprehension online and taps into the immediate and delayed processing of language, was adopted to investigate the dynamics of written irony comprehension.

## Experiment 1: The effects of type of expression (literal vs. ironic) and evaluation (praise vs. criticism) on irony reading

The aim of Experiment 1 was to explore the effects of type of expression (literal vs. ironic) and evaluation (ironic criticism vs. ironic praise) in Chinese written irony comprehension. As the processing of literal statements does not involve inconsistencies between the context and the meaning of the target statement, no significant differences were expected in processing the literal statements as a function of evaluation type (criticism or praise). Conversely, as ironic praise is generally less consistent with the context relative to ironic criticism, significant differences were expected in eye-tracking indicators characterizing the reading process of ironic criticism versus ironic praise.

### Methods

#### Participants

The required sample size for the experiment was calculated using G*power 3.1 software, with a medium effect size set at f = 0.25. At the power level of 0.80 and α level of 0.05, the required number of participants was 24. Finally, 32 college students were randomly recruited, including 27 females (mean age was 20.28 ± 1.49 years old). All participants had normal or corrected to normal vision and were right-h**a**nded. All participants were native speakers of Mandarin Chinese, with English as their second language. The ethical review was approved by the Institutional Review Board of Shandong Normal University. All participants gave their informed consent prior to their inclusion in the study.

#### Experimental design

A 2 (expression type: ironic, literal) × 2 (evaluation type: criticism, praise) within-subjects experimental design was adopted. The dependent variables were the eye-tracking measures of first-pass reading time, regression path reading time, re-reading time, and total reading time while reading the text dialogues, as well as the accuracy in answering the memory and inference questions.

#### Materials

Following Kaakinen et al. (2014), 116 text stories were created, divided into two versions. Each version contained 68 text stories, 24 of which were ironic stories (12 in the criticism and 12 in the praise condition) and 24 were literal condition stories (12 in the praise and 12 in the criticism conditions); twenty filler items were also included (consisting of the same materials in both versions).

**Fig. 1:**
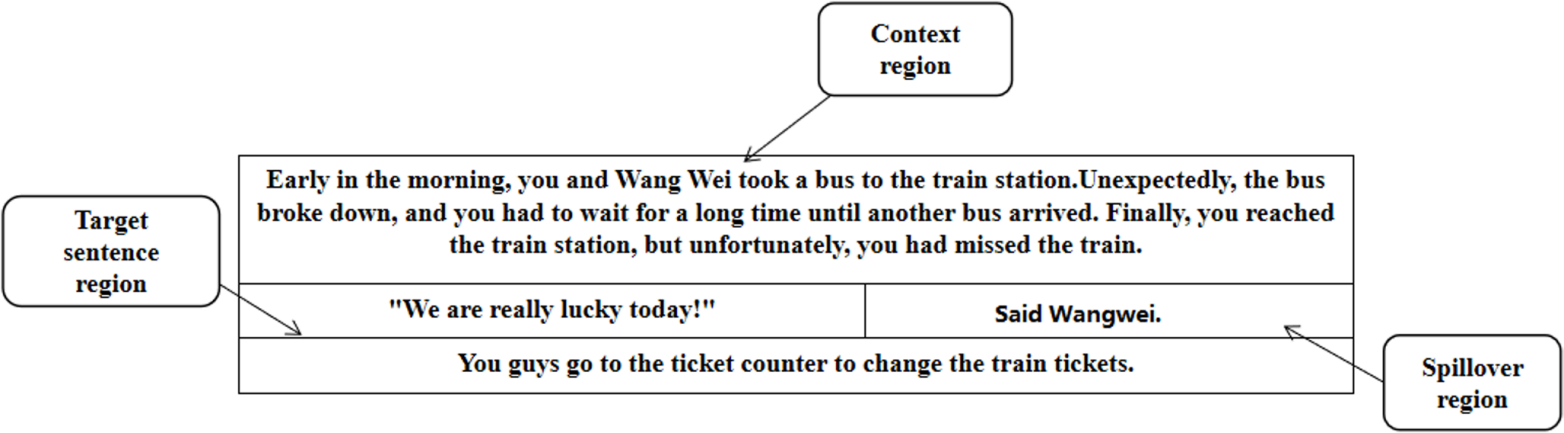
Region of interest division in experiment 1

The reading materials were written in Chinese, involving common scenarios such as movie theaters, dormitories, cafeterias, and parks. Each experimental story consisted of the prior context, target sentence, spillover region (utterance following the target sentence) and story ending (see Fig. 1) (Olkoniemi et al., 2016). The context region, presented in three lines, introduced the dialogue scene and characters. The target sentence consisted of a twelve-word statement, presented in one line, where the speaker used literal / ironic criticism or literal / ironic praise. The spillover region consisted two or three words, such as “Said mum” or “Said Wangwei”. The story ending consisted of a statement to conclude the dialogue. The spillover and the ending regions, which were not relevant to the critical irony processing, were kept identical across the strong and weak context conditions. The context region, target region and the spillover region were analyzed as the regions of interest in revealing effects of irony. Examples of materials for each condition in Experiment 1 are presented in Table 1. The Chinese version of the example materials is presented in Supplementary Table 1.

**Table 1:** A translated example of materials for each condition in experiment 1 (the reading materials were written in Chinese)

	Ironic criticism	Literal criticism	Ironic praise	Literal praise
Context	Early in the morning, you and Wang Wei took a bus to the train station. Unexpectedly, the bus broke down, and you had to wait for a long time until another bus arrived. Finally, you reached the train station, but unfortunately, you had missed the train.	Early in the morning, you and Wang Wei took a bus to the train station. Unexpectedly, the bus broke down, and you had to wait for a long time until another bus arrived. Finally, you reached the train station, but unfortunately, you had missed the train.	You are usually very thrifty and reluctant to spend money on expensive items. However, when it was your good friend’s birthday, you spent a lot of money to buy them a luxury brand handbag.	You are usually very thrifty and reluctant to spend money on expensive items. However, when it was your good friend’s birthday, you spent a lot of money to buy them a luxury brand handbag.
**Target sentence**	“We are really lucky today!” Said Wang Wei.	“We are really unlucky today!” Said Wang Wei.	“You are really stingy!” said the friend.	“You are really generous!” said the friend.
**Ending sentence**	You guys go to the ticket counter to change the train tickets.	You guys go to the ticket counter to change the train tickets.	A friend gave you a big hug.	A friend gave you a big hug.
**Memory question**	You didn’t catch the train.	You didn’t catch the train.	You are a thrifty person.	You are a thrifty person.
**Inference question**	Wang Wei feels that you are unlucky today.	Wang Wei feels that you are unlucky today.	Your friend thinks that you are generous towards her.	Your friend thinks that you are generous towards her.

To ensure the validity of the experimental materials, 24 participants (15 females, average age 20.75 ± 1.80 years) were selected to evaluate the logical coherence, degree of irony, and contextual inconsistency of the irony materials using a five-point Likert scale. These participants did not participate in the formal experiment.

Regarding the evaluation of logical coherence, participants were asked to evaluate whether the dialogues including context text, target text and the ending text were logical or not. Scores from 1 to 5 represented very poor, poor, ordinary, good, and very good logical coherence, respectively. The results showed no significant differences (*F*(3, 24)= 2.137, *p* = .101) in the logical coherence of the sentences among the four conditions: ironic criticism (M = 4.43, SD = 0.11), ironic praise (M = 4.36, SD = 0.12), literal criticism (M = 4.38, SD = 0.08), and literal praise (M = 4.41, SD = 0.07).

Regarding the evaluation of the degree of irony, scores of 1–5 represented very low, low, ordinary, high, and very high, respectively. The results showed that the score of ironic criticism (M = 4.59, SD = 0.26) was significantly higher than that of literal criticism (M = 1.29, SD = 0.22; *t* (46) = -45.32, *p* < .001), and the score of ironic praise (M = 4.46, SD = 0.10) was significantly higher than that of literal praise (M = 1.34, SD = 0.26; *t* (46) = -52.58, *p* < .001). This indicates that the ironic materials in this experiment effectively convey the meaning of irony. There was no significant difference between the scores of ironic criticism (M = 4.62, SD = 0.26) and ironic praise (M = 4.37, SD = 0.29; *t* (46) = 1.98, *p* = .055). To eliminate the possible influence of the degree of irony on the processing time of ironic criticism and ironic praise, the degree of irony was used as a covariate in the subsequent eye movement analyses.

Regarding the evaluation of contextual inconsistency in ironic materials, scores of 1–5 represented very low, low, ordinary, high, and very high levels of contextual inconsistency, respectively. The results showed no significant difference in contextual inconsistency between ironic criticism (M = 4.28, SD = 0.20) and ironic praise (M = 4.18, SD = 0.29; *t* (46) = 1.424, *p* = .16). According to the Restriction-Satisfaction Model (Read & Miller, 1998), context can affect the reading of ironic texts. Yet, the lack of significant difference in contextual inconsistency can eliminate the influence of context on the processing of the two types of irony.

#### Apparatus and procedure

An Eyelink 1000 + eye-tracker (SR Research, Canada) with a sampling rate of 1000 Hz was used. Stimuli were presented on a 19-inch CRT monitor (120 Hz refresh rate, 1024 × 768 resolution).

Participants stabilized their head using a chin and forehead rest. They completed 12 practice trials before advancing to the main experiment after reaching 70% accuracy. The main experiment consisted of 68 trials, with a mid-session break.

**Fig. 2:**
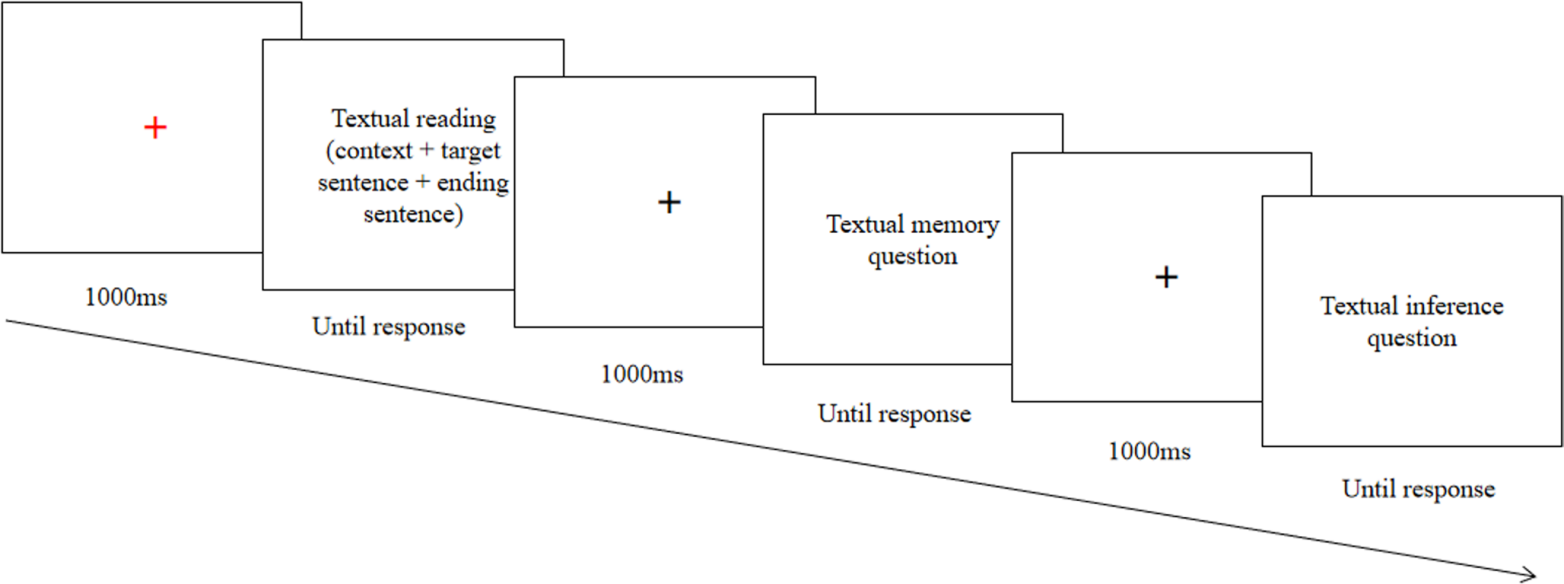
The experimental procedure for each trial in Exp. 1

Each trial began with a 1000ms central red fixation cross (“+”), followed by a text. Participants self-paced their reading. After reading, participants pressed the “O” key to proceed, then viewed a black fixation cross for 1000ms. Following each story, two comprehension questions were presented in statement format: one tested memory for content, the other assessed inference ability regarding ironic meaning and speaker’s mental state. Half of the statements were correct. Participants responded with “F” (correct) or “J” (incorrect). It took about 30 min to complete the session. The trial structure was illustrated in Fig. 2.

#### Data analysis

Two participants’ data were excluded because the eye movement drift was greater than the acceptable range. The final sample size was 30 participants (4 males, 26 females). A total of 1,440 trials (30 participants × 48 trials) were initially recorded in Experiment 1. We deleted the data with fixation durations less than 80ms or greater than 1200ms (Yan et al., 2017), and removed data points that were three standard deviations beyond overall grand mean. Data preprocessing was performed separately for each region of interest (ROI) and for each eye-tracking measure because data loss (e.g., due to blinks) can occur independently both for spatial regions and dependent measures. Following these procedures, the numbers of valid trials retained for analysis across the four eye-tracking measures ranged from 1,366 to 1,416 (94.86% – 98.33%) for the Context Region, from 1,334 to 1,418 (92.64% – 98.47%) for the Target Sentence Region, and from 1,269 to 1,275 (88.13% – 88.54%) for the Spillover Region.

Each text material was divided into four regions (Fig. 1): the context region, the target sentence region, spillover region and the ending region (Olkoniemi et al., 2016). Four eye movement measures were selected as indices of the time course of processing. (1) First-pass reading time sums up all the fixations made on an area of interest when first encountering it and before moving away from it either to a previous or subsequent text region. It was used to index the immediate comprehension. (2) Regression path reading time refers to the sum of the duration of all fixations during the period when the participant’s gaze enters the area of interest and before it exits to the next area of interest. It differs from first-pass reading time in that it also includes look-backs to previous regions of interest. Thus, the measure also reflects the process of integrating the target region with the prior context. (3) Rereading time refers to the duration of the regression path reading time for an area of interest subtracted from its first-pass reading time. Thus, this measure is a purer measure of later integrative processing than regression path reading time, as it only includes rereading fixations. It reflects the process in which the participant encounters a comprehension difficulty leading to rereading of an area of interest (Yan et al., 2017). (4) Total reading time refers to the total duration of the participant’s gaze on an area of interest (first-pass reading + later rereading), which is sensitive to cognitive processing requiring overall more time or effort (Holmqvist et al., 2011). The four eye movements parameters comprehensively describe the timeline of written irony processing from early to late reading stages. Specifically, it is possible to tease apart the time course of irony processing, that is, whether the effect emerges already during the immediate encounter with the text information (indexed by first-pass reading time) or only with some delay (indexed by the other measures).

The R statistical software (Version 4.1.2; R Core Team, 2021) and linear mixed-effects models (LMMs; Baayen et al., 2008) were used to analyze the data. To eliminate the inherent positive skewness and non-normal distribution of the fixation time data, we log-transformed the data of each eye movement measure. Only trials with correct responses to the comprehension questions were included in the analysis.

To supplement and validate the results from the LMMs and to evaluate the strength of evidence for the observed effects, we conducted complementary Bayesian analyses using the brms package (Bürkner, 2017, 2018) in the R environment. The Bayesian models maintained an identical structure to their LMM counterparts. For the fixed-effects slope coefficients, we specified a weakly informative normal prior–Normal (0, 0.5). This scale was informed by the range of typical effect sizes in psycholinguistic research, providing sufficient regularization without unduly constraining parameter estimates. For the model intercept, a broader normal prior – Normal (0, 5) was used to accommodate the grand mean of the log-transformed reaction time data. For all other model parameters (e.g., standard deviations of random effects and the residual), we used the default prior distributions provided by the brms package.

All models were explicitly defined using formula notation. For instance, the model for first-pass reading time (FPRT) in the target region of Experiment 1 was specified as following:

$$\\\begin{array}{c}\log(FPRT)\sim ExpressionType\times EvaluationType+\\IronyDegree+(\left.1\right|Subject)+(\left.1\right|Item)\end{array}\\$$

Each model was run with four independent Markov Chain Monte Carlo (MCMC) sampling chains. Each chain underwent 8,000 iterations, with the first 4,000 iterations discarded as warm-up, resulting in a total of 16,000 valid posterior samples retained for analysis. The convergence of the models was excellent, as indicated by all parameters having Rhat values equal to or very close to 1.00 (specifically, all Rhat values < 1.01), and effective sample sizes (ESS) substantially exceeding 1,000, demonstrating that the sampling was sufficient and the models had converged well. The existence of effects was evaluated by examining whether the 95% Highest Density Intervals (HDI) included zero. An effect was considered credible if the 95%HDI did not include zero. Additionally, we computed Bayes Factors (*BF*_01_) via bridge sampling to quantify the strength of evidence in the data supporting the null hypothesis (H_0_, indicating the absence of an effect) relative to the alternative hypothesis (H_1_, indicating the presence of an effect). Specifically, *BF*₀₁ > 1 indicates evidence in favor of H₀, while *BF*₀₁ < 1 indicates evidence in favor of H₁ (Jeffreys, 1961).

#### Preregistration

The study design and hypotheses were preregistered on Open Science Framework (OSF) prior to data collection: https://osf.io/hpfv7?mode=&revisionId=&view_only=.

### Results

#### Eye-tracking results

The descriptive statistics for the first-pass reading time, regressions path time, total reading time, and rereading time in the context region, the target sentence region and the spillover region are shown in Table 2.

**Table 2:** Means and standard deviations (in parentheses) of the eye-tracking measures (in ms) in experiment 1.

ROI		Irony		Literal	
		Criticism	Praise	Criticism	Praise
**Context region**	First-pass reading time	2998 (1411)	2994 (1335)	2900 (1283)	2891 (1242)
	Regression path reading time	5049 (1996)	5558 (2305)	5089 (2067)	4916 (1832)
	Rereading time	2123 (1279)	2573 (1486)	2215 (1311)	2130 (1247)
	Total reading time	3442 (1596)	3612 (1691)	3406 (1650)	3325 (1509)
**Target sentence region**	First-pass reading time	533 (282)	628 (353)	543 (278)	554 (301)
	Regression path reading time	2258 (1507)	2658 (1723)	2261 (1474)	2228 (1430)
	Rereading time	1802 (1481)	2149 (1803)	1768 (1441)	1738 (1452)
	Total reading time	962 (528)	1196 (646)	957 (499)	980 (497)
**Spillover region**	First-pass reading time	213 (86)	218 (79)	213 (91)	202 (72)
	Regression path reading time	1413 (1169)	1579 (1264)	1439 (1128)	1359 (1064)
	Rereading time	1285 (1139)	1472 (1270)	1346 (1076)	1283 (1022)
	Total reading time	280 (164)	295 (174)	285 (177)	254 (145)

LMMs were conducted on the first-pass reading time, regression path reading time, re-reading time, and total reading time for the context region, the target region and the spillover region. In the linear mixed modal, expression type (ironic, literal), evaluation type (criticism, praise) and the interaction between them were entered as fixed effects, the subjects and items were entered as random effects. In addition, degree of irony was used as a covariate to be controlled in the model. The results showed that the covariate (i.e., degree of irony) was not significant in the models for the different eye-tracking measures in the context, target and spillover regions (all *ps* > 0.05). The statistical power of degree of irony in the above-mentioned models is presented in the Supplementary Tables 2–4.

To ensure the reliability of the results from the linear mixed-effects models, we computed variance inflation factors (VIF) for all fixed effects to assess multicollinearity. The results showed that the vast majority of VIF values were below 5 (range: 1.016–1.467), indicating an absence of significant multicollinearity and thus ensuring the reliability of parameter estimates (O’Brien, 2007). We observed a specific exception in the first-pass reading time (FPRT) measure for the target region, where the VIF values for irony degree and expression type were elevated (up to 6.29). This is a theoretically expected pattern, as the rating of degree of irony is an intrinsic property of the ironic stimuli relative to literal stimuli. Despite this local collinearity, the irony degree covariate was not significant in any model (all *p*s > 0.05). The inclusion of irony degree did not alter the significance patterns of the key effects (i.e., the interaction between expression and evaluation type). Therefore, the parameter estimates for our effects of theoretical interest are considered reliable and interpretable. The details of VIF for each factor of Experiment 1 are shown in the Supplementary Table 5. The statistical results for the target region are shown in Table 3.

**Table 3:** The results of the LMM and bayesian analysis for the different eye-tracking measures in the target sentence region in experiment 1

	b	SE	t	*p*	95% HDI	BF_01_
	First-pass reading time					
Expression type(ironic vs. literal language)	-0.038	0.075	-0.504	0.615	[-0.220, 0.300]	3.793
Evaluation type(criticism vs. praise)	-0.060	0.030	-1.923	0.055	[-0.120, 0.050]	8.361
Expression type × Evaluation type	0.130	0.060	2.156	0.031*	**[0.003**,** 0.300]**	**0.729**
	Simple effects analysis					
Irony: criticism vs. praise	-0.123	0.043	-2.853	0.005**	**[0.018**,**0.220]**	
Literal language: criticism vs. praise	-0.007	0.043	-0.167	0.868	[-0.150, 0.089]	
	**Regression path reading time**					
Expression type(ironic vs. literal language)	-0.072	0.072	-1.007	0.314	[-0.360, 0.050]	1.135
Evaluation type(criticism vs. praise)	-0.084	0.043	-1.942	0.054	[-0.140, 0.020]	4.274
Expression type × Evaluation type	0.179	0.088	2.026	0.045*	**[0.002**,** 0.290]**	**0.793**
	Simple effects analysis					
Irony: criticism vs. praise	-0.173	0.063	-2.026	0.006**	**[0.008**,** 0.233]**	
Literal language: criticism vs. praise	0.006	0.061	0.093	0.926	[-0.125, 0.097]	
	**Re-reading time**					
Expression type(Ironic vs. literal language)	-0.126	0.143	-0.879	0.379	[-0.510, 0.020]	1.913
Evaluation type(criticism vs. praise)	-0.062	0.069	-0.893	0.374	[-0.150, 0.050]	5.050
Expression type × Evaluation type	0.169	0.139	1.216	0.226	[-0.050, 0.350]	1.610
	**Total reading time**					
Expression type(ironic vs. literal language)	-0.043	0.065	-0.669	0.504	[-0.370, 0.000]	1.614
Evaluation type(criticism vs. praise)	-0.111	0.037	-3.023	0.003**	**[-0.170**,**-0.040]**	**0.093**
Expression type × Evaluation type	0.181	0.075	2.422	0.017*	**[0.020**,** 0.250]**	**0.410**
	Simple effects analysis					
Irony: criticism vs. praise	-0.201	0.053	-3.801	<0.001***	**[0.072**,** 0.261]**	
Literal language: criticism vs. praise	0.021	0.052	0.394	0.694	[-0.125, 0.097]	

Bold font indicates significant results. * *p* < .05, ** *p* < .01, *** *p* < .001. For Bayesian analysis, *BF*_*01*_ < 1 indicates evidence for the alternative hypothesis (H₁); *BF*_*01*_ > 1 indicates evidence for the null hypothesis (H_0_)

A significant interaction between Expression type and Evaluation type was observed across three reading time measures including first-pass reading time, regression path reading time and total reading time. The Bayesian model comparison converged with these findings, providing evidence for the interaction in each measure.

**Fig. 3:**
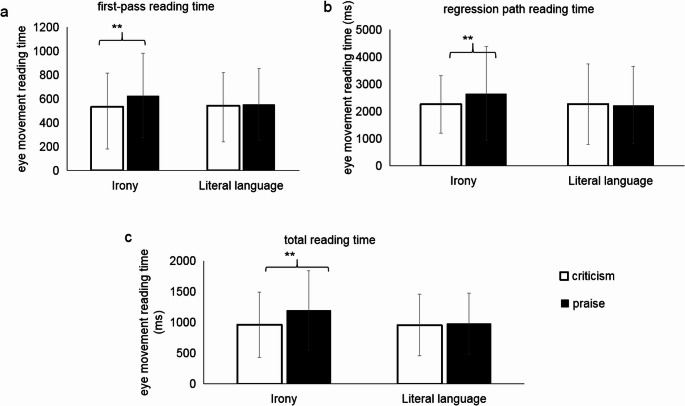
**a**: First-pass reading time; **b**: regression path reading time; and **c**: total reading time of the target sentences in Experiment 1. ** = *p* < .01

For the first-pass reading time, the interaction between expression type and evaluation type was significant in both LMM and Bayesian analyses (LMM: *t* = 2.16, *p* < .05; Bayesian: HDI = [0.003, 0.30], *BF*₀₁ = 0.73). Simple effects analysis revealed significantly longer first-pass reading times for ironic praise (M = 628ms, SD = 353) compared to ironic criticism (M = 533ms, SD = 282; LMM: *t* = -2.85, *p* < .01; Bayesian contrast: Δlog = 0.10, HDI = [0.02, 0.22]), whereas no significant difference was found between literal criticism (M = 543ms, SD = 278) and literal praise (M = 554ms, SD = 301) (Fig. 3a).

Similarly, regression path reading time showed a significant interaction between expression type and evaluation type in both analytical frameworks (LMM: *t* = 2.03, *p* < .05; Bayesian: HDI = [0.002, 0.29], *BF*₀₁ = 0.79). Ironic praise elicited longer regression path reading times (M = 2658ms, SD = 1723) than ironic criticism (M = 2258ms, SD = 1507; LMM: *t* = -2.03, *p* < .01; Bayesian contrast: Δlog = 0.13, HDI = [0.01, 0.23]), while no significant difference emerged between literal criticism (M = 2261ms, SD = 1474) and literal praise (M = 2228ms, SD = 1430) (Fig. 3b).

For total reading time, the main effect of evaluation type was significant in both LMM and Bayesian analyses (LMM: *t* = -3.02, *p* < .05; Bayesian: HDI = [-0.17, -0.04], *BF*₀₁ = 0.09), indicating longer reading times for praise (M = 1086ms, SD = 584) than criticism sentences (M = 959ms, SD = 513). A significant interaction between expression type and evaluation type was also found (LMM: *t* = 2.42, *p* < .05; Bayesian: HDI = [0.02, 0.25], *BF*₀₁ = 0.41). Simple effects analysis demonstrated significantly longer total reading times for ironic praise (M = 1196ms, SD = 646) relative to ironic criticism (M = 962ms, SD = 528; *t* = -3.80, *p* < .01; Bayesian contrast: Δlog = 0.16, HDI = [0.07, 0.26]), with no significant difference between literal criticism (M = 957ms, SD = 499) and literal praise (M = 980ms, SD = 497) (Fig. 3c).

**Fig. 4:**
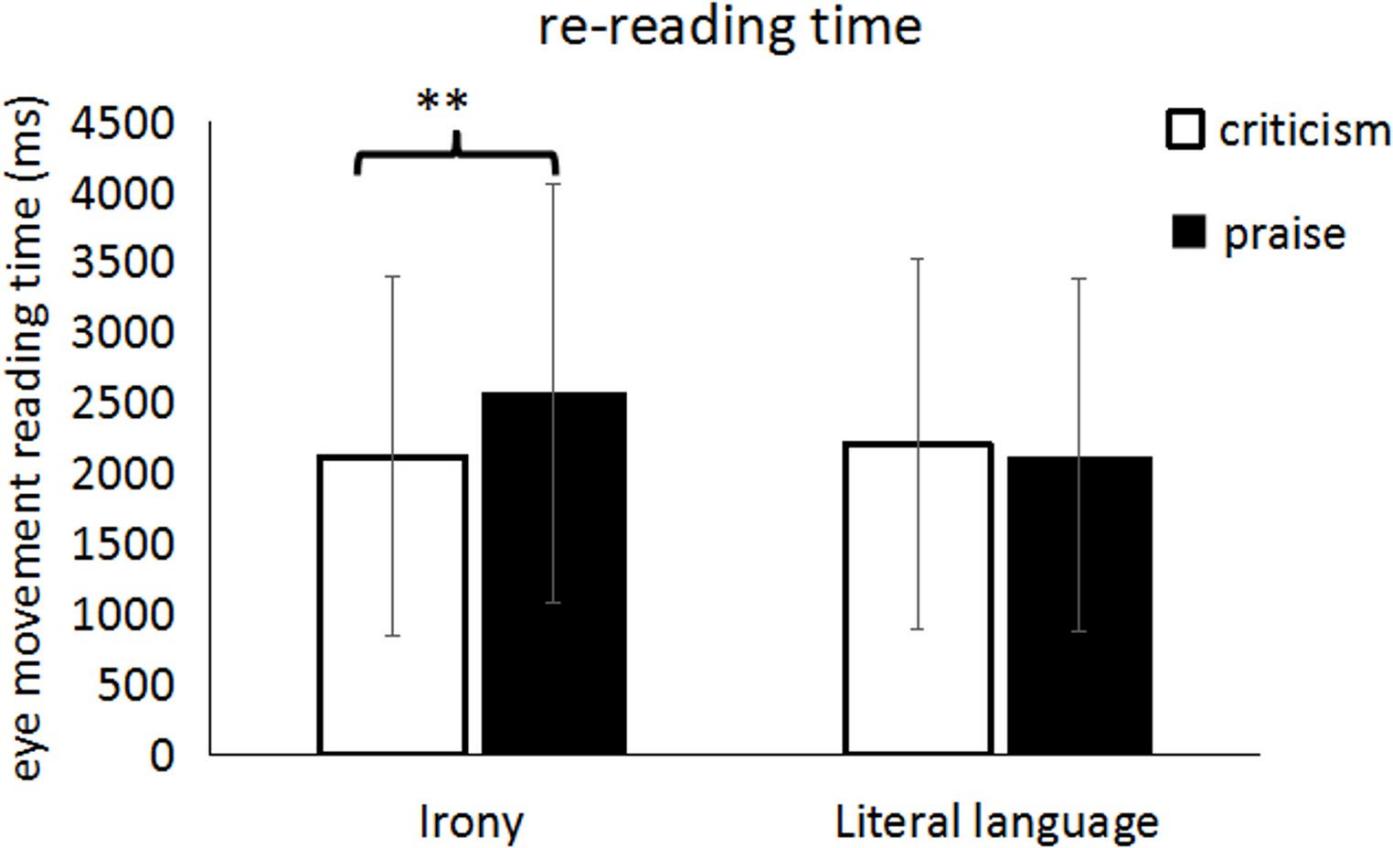
The rereading time of the context region in Experiment 1; ** = *p* < .01

In the context area, the interaction between expression and evaluation type was significant in both LMM and Bayesian analyses for rereading time (LMM: *t* = 2.30, *p* < .05; Bayesian: HDI = [0.002, 0.34], *BF*₀₁ = 0.88). Simple effect analysis found that participants had significantly longer rereading time (2573 ± 1486 ms) for ironic praise compared to ironic criticism (2123 ± 1279 ms; (LMM: *t* = -2.88, *p* < .01; Bayesian contrast: Δlog = 0.13, 95% HDI = [0.01, 0.26]), whereas no significant difference was found between literal criticism and literal praise. There were no significant differences in the other measures for the context area. The results of the LMM and Bayesian analysis for re-reading time in the context area is shown in Table 4; Fig. 4.

For spillover region, no statistically significant effects were found in any dependent measure.

**Table 4:** The results of the LMM and bayesian analysis for re-reading time in the context region in experiment 1

Expression type(ironic vs. literal language)	b	SE	t	*p*	95% HDI	BF_01_
	-0.012	0.091	-0.133	0.894	[-0.290, 0.280]	3.144
Evaluation type(criticism vs. praise)	-0.082	0.045	-1.809	0.073	[-0.130, 0.050]	7.193
Expression type × Evaluation type	0.211	0.091	2.304	0.023*	**[0.001**,** 0.340]**	**0.918**
	Simple effects analysis					
Irony (criticism vs. praise)	-0.187	0.065	-2.879	0.005**	**[0.001**,** 0.250]**	
Literal language (criticism vs. praise)	0.024	0.064	0.371	0.711	[-0.166, 0.079]	

Bold font indicates significant results. * *p* < .05, ** *p* < .01. For Bayesian analysis, *BF*_*01*_ < 1 indicates evidence for the alternative hypothesis (H₁); *BF*_*01*_ > 1 indicates evidence for the null hypothesis (H_0_)

#### Accuracy in responding to memory and inference questions

A 2 (text type: ironic, literal) x 2 (question type: text memory, text inference) x 2 (evaluation type: praise, criticism) repeated measures ANOVA was conducted on performance accuracy. The accuracy results for each condition are shown in Table 5. The results showed a significant main effect of question type (*F*(1, 31) = 10.48, *p* = .003,*η*_*p*_^*2*^ = 0.25), with participants having significantly lower accuracy for inference questions (0.89 ± 0.01) compared to memory questions (0.93 ± 0.01), indicating that text inference questions were more difficult for participants to complete than text memory questions. There were no significant effects for the other variables, indicating that participants were able to complete the experimental task well under all conditions.

**Table 5:** Accuracy for memory and reasoning questions for the different conditions in experiment 1 (M ± SD)

Condition	Ironic criticism	Ironic praise	Literal criticism	Literal praise
Memory	0.92 (0.08)	0.95 (0.06)	0.92 (0.08)	0.92 (0.07)
Inference	0.89 (0.15)	0.91 (0.09)	0.88 (0.11)	0.89 (0.09)

### Discussion

Using a free reading paradigm with eye-tracking, Experiment 1 examined how evaluation type (praise vs. criticism) influences literal and ironic language processing in Chinese. No main effect of expression type was observed, indicating that irony was not processed more slowly than literal statements. This finding contradicts the Standard Pragmatic Model (Grice, 1975; Searle, 1979), which predicts greater processing cost for irony due to sequential meaning reconciliation. Instead, the absence of a significant difference between ironic and literal language aligns with the Direct Access View, which posits early context-lexical interaction and shared mechanisms for literal and figurative processing (Gibbs, 1994).

Critically, ironic praise required significantly longer reading times than ironic criticism across both context and target regions, evident in early (first-pass reading time; Yan et al., 2013) and late (regression path and total reading time; Holmqvist et al., 2011) processing stages. Longer first-pass times on target sentences suggest greater early cognitive demand for ironic praise, while extended late measures reflect continued integration difficulty. Additionally, increased rereading time in the context region for ironic praise indicates deeper contextual reliance during comprehension. These results motivated Experiment 2, which further explored whether and how contextual support modulates the processing of ironic criticism versus ironic praise.

## Experiment 2: Effect of context on processing ironic criticism and ironic praise

The aim of Experiment 2 was to examine whether the degree of inconsistency between the context and the ironic target statement exerts different effects on the cognitive processing of ironic praise and ironic criticism in natural Chinese reading. In the weak inconsistency condition, the inconsistency between the context and the literal meaning of the target utterance is not obvious. Thus, it does not strongly support an ironic interpretation. Hence, it was expected that when the inconsistency is weak the processing time for ironic praise will be significantly longer than that for ironic criticism. In other words, the assumed difficulty in comprehending ironic praise will be observed. In contrast, when the inconsistency between the context and the literal meaning of the target utterance is strong, the ironic interpretation is supported, resulting in no significant difference in processing ironic praise and ironic criticism.

### Methods

#### Participants

The required sample size for the experiment was calculated using G*Power 3.1 software, with a medium effect size set at f = 0.25. At the power level of 0.80 and αlevel of 0.05, the required number of participants was 24. The study adopted a large-sample design to ensure statistical power; finally, thirty-one participants were recruited, including 26 females (20.26 ± 2.05 years old). All participants had normal or corrected-to-normal vision and were right-handed. The language background of the participants was identical to that in Experiment 1. The ethical review was approved by the Institutional Review Board of Shandong Normal University. All participants gave their informed consent prior to their inclusion in the study.

#### Design

A 2 (irony type: ironic criticism vs. ironic praise) × 2 (degree of context inconsistency: strong vs. weak) within-subjects design was adopted in Experiment 2. The dependent variables were the eye-tracking measures of first-pass reading time, regression time, rereading time, and total reading time measured while reading the dialogues, as well as the accuracy in answering the memory and inference questions.

#### Materials

The irony conditions consisted of 116 text segments in total. Among these, 96 segments were distributed across four experimental conditions: ironic praise in a strong context, ironic praise in a weak context, ironic criticism in a strong context, and ironic criticism in a weak context. The 96 experimental items were organized into two blocks. An ABBA counterbalancing procedure was implemented between participants to control for contextual strength and evaluative type (praise vs. criticism), ensuring that each text appeared in only one condition. The remaining 20 segments functioned as filler items. The same filler materials were used in both blocks. One subject read 12 texts in each of the four conditions and the remaining 20 fillers. All four experimental conditions, along with filler items, were presented in a fully randomized sequence. The fillers were structurally matched to the critical ironic stimuli in length and syntactic complexity but contained no ironic or evaluative content. These fillers described literal, everyday scenarios (e.g., *When you woke up in the morning*,* you carefully styled your hair*,* but you didn’t expect the wind to be so strong today. The moment you stepped outside*,* your hair got completely messed up*,* and your classmate’s hat blew away. “The wind is really strong today!” your classmate said.*). The filler materials were inserted in the experiment to balance the experiment: By interspersing neutral passages with ironic ones, we prevented participants from developing a task-specific strategy (e.g., anticipating irony in every trial), thereby promoting naturalistic reading behavior. This approach aligns with established practices in irony research (e.g., Olkoniemi & Kaakinen, 2021; Pexman, 2008), where neutral fillers are critical for controlling pragmatic task effects.

Examples of materials for each condition in Experiment 2 are presented in Table 6. The Chinese version of the example materials is presented in the Supplementary Table 6.

**Table 6:** A translated example of materials for each condition in experiment 2 (the reading materials were written in Chinese)

	Ironic Criticism		Ironic Praise		
	Strong context inconsistency	Weak context inconsistency	Strong context inconsistency	Weak context inconsistency	
**Context**	On the weekend, your family is doing a major cleaning at home. Your mom asks you to clean your own room. You quickly dust off your room with a feather duster, considering it done. However, your room remains messy, with a lot of dust.	On the weekend, your family is doing a major cleaning at home. Your mom asks you to clean your own room. You clean it thoroughly, but when mom comes to inspect, there are still a few books scattered on the floor.	With one month left until summer vacation, you and your fellow villager Xiao Hua have already started booking tickets online. You quickly seize the opportunity and grab a 70% off plane ticket. The total cost is much cheaper than taking a train back home.	With one month left until summer vacation, you and your fellow villager Xiao Hua have already started booking tickets online. You swiftly grab a 30% off plane ticket. The total cost is only slightly more expensive than taking a high-speed train back home.	
**Target sentence**	“You cleaned really well!”	“You cleaned really well!”	“Your plane ticket is really expensive!”	“Your plane ticket is really expensive!”	
**Spillover region**	said Mum	said Mum	said Xiao Hua	said Xiao Hua	
**Ending sentence**	You’re going to wash the dirty clothes too.	You’re going to wash the dirty clothes too.	Xiao Hua keeps snatching tickets online for the trip back home.	Xiao Hua keeps snatching tickets online for the trip back home.	
**Memory question**	You and your mom are cleaning together.	You and your mom are cleaning together.	You and your friend Xiao Hua are booking tickets together.	You and your friend Xiao Hua are booking tickets together.	
**Inference question**	Your mother is not satisfied with the way you cleaned the room.	Your mother is not satisfied with the way you cleaned the room.	Xiao Hua is very envious that you were able to snatch that plane ticket.	Xiao Hua is very envious that you were able to snatch that plane ticket.	

To ensure the validity of the experimental materials, additional 24 participants (15 females and 9 males, aged at 21.00 ± 2.70 years old) were recruited to rate the level of context inconsistency, logical coherence, and degree of irony using a Likert scale ranging from 1 to 5. For the rating of context inconsistency, scores of 1 to 5 represented very low, low, average, high, and very high levels of context inconsistency, respectively. The results showed that for the ironic criticism condition, the ratings of the strong context inconsistency condition (M = 3.76, SD = 0.20) were significantly higher than those of the weak context inconsistency condition (M = 2.47, SD = 0.21, *t*(23) = 20.72, *p* < .001). Similarly, for the ironic praise condition, the ratings of the strong context inconsistency condition (M = 3.97, SD = 0.24) were significantly higher than those for the weak context inconsistency condition (M = 2.70, SD = 0.23, *t*(23) = 23.52, *p* < .001). These results indicate that the manipulation of context inconsistency in the experimental materials was effective.

For the rating of logical coherence, scores of 1 to 5 represented very poor, poor, average, good, and very good logical coherence, respectively. The results showed no significant difference in logical coherence between the strong (M = 3.66, SD = 0.08) and weak (M = 3.71, SD = 0.11) context inconsistency condition for ironic criticism (*t*(23) = -1.67, *p* = .12), or between the strong (M = 3.50, SD = 0.18) and weak (M = 3.55, SD = 0.15) context inconsistency condition for ironic praise (*t*(23) = − 0.19, *p* = .37). These results indicate that the materials had similar logical coherence across all conditions.

For the rating of degree of irony, scores of 1 to 5 represented very low, low, average, high, and very high degrees, respectively. The results showed no significant difference in degree of irony between the strong and weak context inconsistency condition for the ironic praise (strong M = 3.69, SD = 0.14, weak M = 3.67, SD = 0.16, *t*(23) = 0.38, *p* > .05). For the ironic criticism, there was a tendency (*t*(23) = 1.73, *p* = .09) that the degree of irony was greater for the strong (M = 3.81, SD = 0.12) than weak (M = 3.73, SD = 0.19) inconsistency condition. These results indicate that the language materials used in the experiment were effective in conveying ironic meanings in the weak and strong inconsistency condition. In order to exclude the effect of the degree of irony, it was entered as a covariate in the statistical analyses.

#### Apparatus and procedure

The apparatus and procedures were the same as in Experiment 1.

#### Data analysis

The procedure of the data analysis was identical to that in Experiment 1. One participant’s data was excluded because the eye movement drift was greater than the acceptable range. Thus, the final sample size was 30 participants (4 males, 26 females). We employed the same criteria for data exclusion as in Experiment 1. Each participant completed 48 trials, resulting in a total of 1,440 trials for initial processing. The numbers of valid trials retained for analysis varied between 1,408 and 1,415 (97.85% – 98.26%) in the Context Region, between 1,296 and 1,313 (90% – 91.18%) in the Target Sentence Region, and between 1,283 and 1,303 (89.10% – 90.49%) in the Spillover Region across the four eye-tracking measures.

### Results

#### Eye-tracking results

The descriptive statistics were shown in Table 7. It should be noted that the analyses for the context region were first conducted separately for each sentence and then averaged for the sentences making up the context region.

**Table 7:** Means and standard deviations (in parentheses) of the eye-tracking measures (in ms) for the regions of interest (ROIs) in experiment 2

ROI		Strong context inconsistency		Weak context inconsistency	
		Ironic criticism	Ironic praise	Ironic criticism	Ironic praise
**Context region**	First-pass reading time	2873 (1305)	2974 (1262)	2888 (1166)	2933 (1185)
	Regression path reading time	5727 (2497)	5895 (2364)	5646 (2223)	6235 (2719)
	Rereading time	2730 (1668)	2875 (1677)	2647 (1530)	3159 (1977)
	Total reading time	3739 (1837)	3796 (1819)	3685 (1557)	3950 (1785)
**Target sentence region**	First-pass reading time	499 (299)	507 (287)	527 (311)	612 (371)
	Regression path reading time	3569 (2370)	3308 (2067)	2935 (1770)	3906 (2402)
	Rereading time	3044 (2444)	2838 (2159)	2399 (1837)	3263 (2455)
	Total reading time	1161 (672)	1241 (695)	1083 (610)	1410 (821)
**Spillover region**	First-pass reading time	195 (61)	202 (62)	196 (58)	203 (66)
	Regression path reading time	1699 (1396)	1816 (1385)	1691 (1309)	2067 (1682)
	Rereading time	1557 (1383)	1650 (1370)	1536 (1287)	1957 (1661)
	Total reading time	232 (116)	252 (126)	239 (118)	257 (132)

LMM models were computed for the context region and the target sentence region. In the linear mixed modal, irony type (criticism vs. praise), context inconsistency (strong vs. weak) and the interaction between them were entered as fixed effects, the subjects and items were entered as random effects. Degree of irony was used as a covariate to be controlled in the model, eliminating its potential effect with the irony type and context inconsistency on irony comprehension. The results showed that the covariate (i.e., degree of irony) was not significant in the models for the different eye-tracking measures in the context, target and spillover regions (all *ps* > 0.05). The statistical power of degree of irony in the above-mentioned models are presented in the Supplementary Tables 7–9. To ensure the reliability of the results from the linear mixed-effects model, we computed variance inflation factors (VIF) for all fixed effects to assess multicollinearity. The results showed that all VIF values were below 5 (range: 1.016–1.467), indicating an absence of significant multicollinearity and thus ensuring the reliability of parameter estimates (O’Brien, 2007). The details of VIF for each factor of Experiment 2 are shown in the Supplementary Table 10. The statistical results for the target region are shown in Table 8.

**Table 8:** The linear mixed-effects model analysis and bayesian analysis for the different eye-tracking measures in the target sentence region in experiment 2

	b	SE	t	*p*	95% HDI	BF_01_
	First-pass reading time					
Irony type: Criticism vs. Praise	-0.055	0.033	-1.685	0.092	[-0.150, 0.020]	4.509
Context inconsistency: Strong vs. Weak	-0.67	0.036	-1.845	0.065	[-0.030, 0.150]	4.713
**Irony type × Context inconsistency**	**-0.146**	**0.063**	**-2.313**	0.021*	**[-0.370**,** -0.050]**	**0.241**
	Simple effects analysis					
Strong context inconsistency: Ironic criticism vs. Ironic praise	0.018	0.046	0.390	0.698	[− 0.158, 0.077]	
**Weak context inconsistency: Ironic criticism vs. Ironic praise**	**0.128**	**0.045**	**2.851**	0.005**	**[0.062**,** 0.286]**	
	Regression path reading time					
Irony type: Criticism vs. Praise	-0.074	0.055	-1.326	0.188	[-0.150, 0.030]	4.049
Context inconsistency: Strong vs. Weak	0.030	0.062	0.479	0.633	[-0.140, 0.060]	7.700
**Irony type × Context inconsistency**	**-0.285**	**0.107**	**-2.662**	0.009**	**[-0.470**,** -0.120]**	**0.028**
	Simple effects analysis					
Strong inconsistency: Ironic criticism vs. Ironic praise	-0.069	0.078	-0.878	0.382	[− 0.215, 0.040]	
**Weak context inconsistency: Ironic criticism vs. Ironic praise**	**0.216**	**0.076**	**2.852**	0.005**	**[0.083**,** 0.329]**	
	Rereading time					
Irony Type: Criticism vs. Praise	-0.085	0.072	-1.158	0.250	[-0.150, 0.050]	4.831
Context inconsistency: Strong vs. Weak	0.070	0.082	0.849	0.398	[-0.160, 0.060]	6.237
**Irony type × Context inconsistency**	**-0.321**	**0.142**	**-2.258**	0.026*	**[-0.490**,** -0.110]**	**0.059**
	Simple effects analysis					
Strong context inconsistency: Ironic criticism vs. Ironic praise	0.075	0.104	0.722	0.472	[− 0.243, 0.043]	
**Weak context inconsistency: Ironic criticism vs. Ironic praise**	**-0.246**	**0.101**	**2.443**	0.017*	**[0.063**,** 0.340]**	
	Total reading time					
**Irony type: Criticism vs. Praise**	**-0.140**	**0.048**	**-2.950**	0.004**	**[-0.220**,** -0.040]**	**0.145**
Context inconsistency: Strong vs. Weak	0.001	0.053	0.021	0.983	[-0.110, 0.100]	8.693
Irony type × Context Inconsistency	-0.132	0.092	-1.446	0.152	[-0.310, 0.030]	1.468

Bold font indicates significant results. * = *p* < .05, ** = *p* < .01. For Bayesian analysis, *BF*_*01*_ < 1 indicates evidence for the alternative hypothesis (H₁); *BF*_*01*_ > 1 indicates evidence for the null hypothesis (H_0_)

A significant interaction between irony type and degree of context inconsistency was observed across three reading time measures for the target sentence. The Bayesian model comparison converged with these findings, providing evidence for the interaction in each measure.

**Fig. 5:**
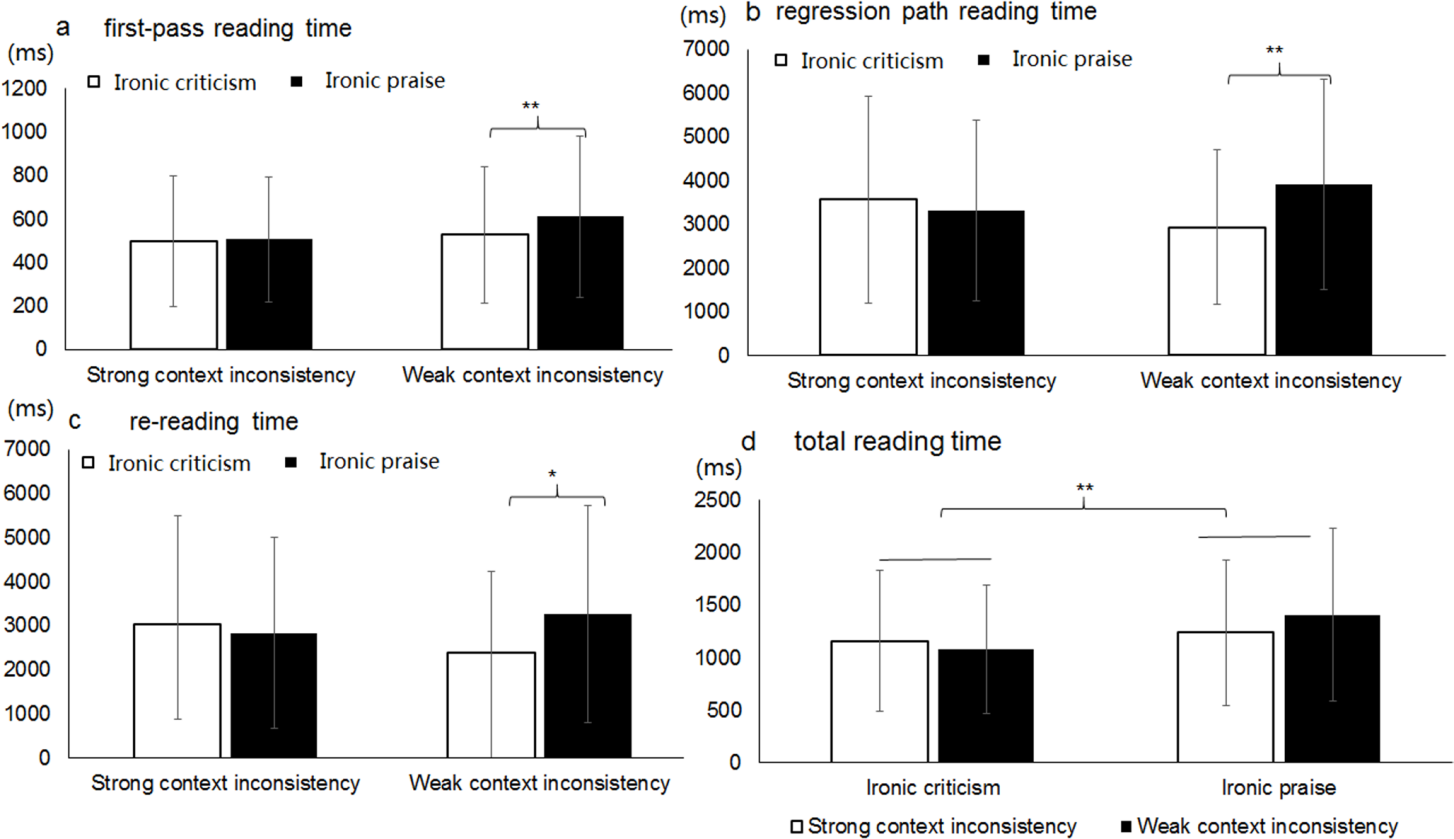
The means observed for the target sentence as a function of irony type and degree of context inconsistency in Experiment 2. The unit of vertical axis is millisecond and the error bars denote standard deviations. Panel a shows the results for first-pass reading time, Panel b for regression path reading time, Panel c for re-reading time, and Panel d for total reading time. Note: * = *p* < .05, ** = *p* < .01

For first-pass reading time, the interaction effect was significant (*t* = -2.31, *p* < .05), ironic praise (612 ± 371 ms) elicited significantly longer reading times than ironic criticism (527 ± 311 ms) under weak context inconsistency (*t* = 2.85, *p* < .01), while no significant difference was found between ironic praise (507 ± 287 ms) and ironic criticism (499 ± 299 ms) in the strong inconsistency condition (*t* = 0.39, *p* > .05; Fig. 5a). Consistently, the Bayesian simple-effects contrast showed longer first-pass reading times for praise than criticism under weak inconsistency (Δlog = 0.17, HDI [0.06, 0.29]), but not under strong inconsistency (Δlog = − 0.04, HDI [− 0.16, 0.08]).

Similarly, an interaction was also observed in regression path time (*t* = -2.66, *p* < .01); ironic praise (3906 ± 2402 ms) required significantly more time than ironic criticism (2935 ± 1770 ms) in the weak inconsistency condition (t = 2.85, *p* < .01), whereas no significant difference emerged between ironic praise (3308 ± 2067 ms) and ironic criticism (3569 ± 2370 ms) in the strong inconsistency condition (*t* = − 0.88, *p* > .05; Fig. 5b). The Bayesian simple effects paralleled this pattern (weak: Δlog = 0.20, HDI [0.08, 0.33]; strong: Δlog = − 0.10, HDI [− 0.22, 0.04]).

The same pattern held for rereading time (*t* = -2.26, *p* < .05), with ironic praise (3263 ± 2455 ms) taking significantly longer to reread than ironic criticism (2399 ± 1837 ms) in the weak inconsistency condition (*t* = 2.44, *p* < .05), but no significant difference emerged between ironic praise (2838 ± 2159 ms) and ironic criticism (3044 ± 2444 ms) under strong inconsistency (*t* = 0.72, *p* > .05; Fig. 5c). Bayesian simple effects again supported a significant difference between ironic praise and criticism only under weak inconsistency (weak: Δlog = 0.20, HDI [0.06, 0.34]; strong: Δlog = − 0.09, HDI [− 0.24, 0.04]).

Finally, there was a significant main effect of irony type for total reading time of the target sentences (*t* = -2.95, *p* < .01; see Fig. 5d) and Bayesian analysis corroborated this main effect (*BF*_01_= 0.15; HDI [− 0.22, − 0.04]). Participants spent significantly more time reading the ironic praise (1326 ± 765 ms) than the ironic criticism (1123 ± 643 ms) utterances.

Overall, the results consistently showed that ironic praise required longer processing times than ironic criticism only in the weak context inconsistency condition, with no significant differences observed under strong inconsistency.

For both the context and spillover regions, no statistically significant effects were found in any dependent measure by LMM, a pattern that was supported by the Bayesian model comparison. The Bayesian results for the context and spillover regions are shown in the Supplementary Tables 11 and 12.

#### Accuracy in responding to memory and inference questions

A three-way repeated-measures ANOVA was conducted on the accuracy in responding to the questions with the factors of irony type (ironic criticism, ironic praise), degree of context inconsistency (strong, weak), and question type (text memory, text inference). The accuracy results for each condition are presented in Table 9. The results showed a significant main effect of context inconsistency, *F*(1, 30) = 36.43, *p* < .001, *η*_*p*_^*2*^ = 0.55), with higher accuracy for the strong context inconsistency (0.92 ± 0.09) than the weak context inconsistency condition (0.86 ± 0.10), indicating that strong context inconsistency provided more information and could help improve participants’ text inference and memory performance. All other effects were non-significant.

**Table 9:** Accuracy (M ± SD) in responding to memory and inference questions as a function of irony type and context inconsistency in experiment 2

	Strong Context Inconsistency		Weak Context Inconsistency	
	Ironic criticism	Ironic praise	Ironic criticism	Ironic praise
Memory	0.94 (0.07)	0.92 (0.07)	0.91 (0.09)	0.86 (0.09)
Inference	0.91 (0.09)	0.91 (0.11)	0.84 (0.10)	0.84 (0.13)

### Discussion

In Experiment 2, we manipulated contextual inconsistency (strong vs. weak) to examine its effect on the processing of ironic criticism versus ironic praise. The results showed that ironic praise required significantly longer total reading time than ironic criticism, indicating greater cognitive demand. More importantly, a significant interaction between inconsistency strength and irony type emerged: under weak inconsistency, ironic praise elicited longer first-pass reading, regression path, and rereading times compared to ironic criticism, whereas under strong inconsistency, no such differences were observed. This pattern aligns with prior findings (Rivière et al., 2018), suggesting that unclear contextual support increases the difficulty of interpreting ironic praise, whereas strong inconsistency provides clear cues for ironic interpretation, thereby eliminating processing differences between the two irony types. The time course of effects revealed both early (first-pass) and late (regressions, rereading) processing difficulties for ironic praise in weak contexts, suggesting increased immediate attention and subsequent contextual reinspection in processing ironic praise. Comprehension accuracy was also higher in the strong inconsistency condition, supporting the view that clear contextual mismatch aids irony interpretation and text memory.

Previous research has shown that working memory capacity (WMC) modulates the time course of irony comprehension, with high-WMC readers resolving irony faster than low-WMC readers (Kaakinen et al., 2014; Olkoniemi et al., 2016, 2019). However, these studies focused on ironic criticism. Whether WMC similarly affects the more demanding comprehension of ironic praise remains unclear. To address this, Experiment 3 employed the weak inconsistency condition—which elicits divergent processing for ironic praise versus criticism—to investigate the role of WMC in irony comprehension.

## Experiment 3: Effect of working memory capacity on processing ironic criticism and ironic praise under weak context inconsistency

The aim of Experiment 3 was to explore whether individual differences in working memory capacity affect the processing of ironic praise and ironic criticism in Chinese reading under weak context inconsistency, and to explore the time course of their influence. It was expected that participants with low WMC may show difficulty in reading ironic praise relative to ironic criticism. In contrast, participants with high WMC may be able to solve the cognitive processing difficulty inherent in ironic praise; thus, for them no difference would be found between the two irony conditions.

### Methods

#### Participants

One hundred and eight undergraduate students (90 females, aged 20.96 ± 1.57 years) were recruited to participate in the working memory capacity test (WMC). WMC was estimated with the Chinese translation version of the Reading Span test originally developed by Daneman and Carpenter (1980). Participants were presented with several sets of sentences and were asked to judge whether each sentence was logically true or false after reading it. After each set of sentences, participants were also required to type the final words of each sentence in the order that the sentences were presented. The number of sentences in each set increased from 2 to 6, and each set was presented for three times. The average accuracy rate was recorded. If the participant could correctly judge the sentences and recall the final words of all the sentences in two or more of the three presentations of set-size 2, their working memory capacity was classified as 2 and they moved on to the next set with more sentences. If they could only do it in one of the three presentations, their working memory capacity was classified as 1.5. If all three presentations were incorrect, their working memory capacity was classified as 1, and they did not move on to the next set. The maximum score for the working memory capacity test was 6. This was achieved if the participant was able to correctly judge the 6 sentences and recall the final words of all the sentences in two or three presentations of set-size 6.

Based on the test, 28 participants (26 females, mean score of 5.07, SD = 0.58), who scored at the top 27% in the WMC test, were assigned to the high WMC group while another 28 participants (21 females, mean score of 2.80, SD = 0.60), who scored at the bottom 27% in the WMC test, were assigned to the low WMC group. An extreme group comparison was used to maximize the chances to obtain an effect of WMC on irony comprehension. The language background of the participants was identical to that in Experiment 1. All participants gave their informed consent prior to their inclusion in the study.

#### Design and apparatus

A 2 (irony type: ironic praise vs. ironic criticism) × 2 (WMC: high vs. low) mixed design was adopted, with irony type as a within-subject factor and WMC as a between-subjects factor. The dependent variables and the apparatus were the same as in Experiment 1.

#### Materials

Twenty-four ironic criticism and 24 ironic praise texts under weak context inconsistency from Experiment 2 were used as the materials, together with 20 filler texts.

#### Procedure

The procedure of the reading experiment was identical to that in Experiment 1.

#### Data analysis

The data analysis procedure and the criteria for data exclusion were identical to those in Experiment 1. Data from 56 participants who each completed 48 experimental trials resulted in 2,688 total trials for preprocessing. For each region of analysis, valid trials accounted for 97.88% – 98.44% (2,631 to 2,646) of the context, 97.02% – 98.18% (2,608 to 2,639) of the target sentence, and 86.7% – 89.47% (2,322 to 2,405) of the spillover region.

### Results

#### Eye-tracking results

Descriptive statistics of the first-pass time, regression path time, total reading time, and rereading time for the context region, the target sentence region and the spillover region are shown in Table 10.

**Table 10:** Means and standard deviations (in parentheses) of the eye-tracking measures (ms) in experiment 3

ROIs		High working memory capacity		Low working memory capacity		
		Ironic Criticism	Ironic Praise	Ironic Criticism		Ironic Praise
Context region	First-pass reading time	3110 (1422)	3072 (1402)	3482 (1519)		3402 (1582)
	Regression path reading time	6100 (2670)	6229 (2552)	6993 (2904)		7490 (3041)
	Rereading time	2908 (1930)	3142 ((Colston, and O’Brien, 1994))	3409 (2274)		3963 (2431)
	Total reading time	3977 (1902)	3929 (1869)	4583 (2026)		4668 (2161)
Target sentence region	First-pass reading time	583 (367)	646 (434)	542 (372)		622 (449)
	Regression path reading time	3699 (2639)	3810 (2519)	4196 (3134)		4905 (3439)
	Rereading time	3152 (2711)	3186 (2635)	3660 (3255)		4299 (3581)
	Total reading time	1326 (797)	1495 (808)	1399 (895)		1728 (1007)
Spillover region	First-pass reading time	216 (95)	215 (85)	212 (79)		218 (83)
	Regression path reading time	1970 (1681)	2001 (1643)	2093 (1850)		2615 (2147)
	Rereading time	1818 (1647)	1839 (1597)	1968 (1824)		2486 (2180)
	Total reading time	280 (174)	269 (157)	269 (155)		299 (171)

LMM analyses were conducted on the first-pass reading time, regression path time, rereading time, and total reading time for the context region, the target sentence region and the spillover region. In the LMM, the irony type (criticism vs. praise), working memory capacity (high vs. low) and the interaction between them were entered as fixed effects, the subjects and items were entered as random effects. Degree of irony was used as a covariate to be controlled in the model. The results showed that the degree of irony as a covariate was not significant in the models for the different eye-tracking measures in the context, target and spillover regions (all *ps* > 0.05). The statistical power of degree of irony in the above-mentioned models are presented in the Supplementary Tables 13–15. VIFs were computed to assess multicollinearity among fixed effects predictors. All VIF values (see the Supplementary Table 16 for details) were below the critical threshold of 5 (range: 1.000-1.018), confirming the absence of significant multicollinearity (O’Brien, 2007). The results of the LMM analyses for the target sentence region are shown in Table 11.

**Table 11:** The results of the linear mixed-effects model analysis and bayesian analysis for the various eye-tracking measures in the target sentence region in experiment 3

Conditions	b	SE	t	*p*	95% HDI	BF_01_
	First-pass reading time					
WMC(high vs. low)	-0.089	0.077	-1.154	0.254	[-0.230, 0.070]	3.554
**Irony type (criticism vs. praise)**	**0.081**	**0.031**	**2.627**	0.012*	**[-0.140**,** -0.020]**	**0.661**
Irony type×WMC	-0.028	0.048	-0.573	0.566	[-0.120, 0.070]	8.965
	Regression path reading time					
WMC(high vs. low)	0.145	0.127	1.149	0.255	[-0.110, 0.380]	2.263
Irony type (criticism vs. praise)	-0.112	0.060	-1.853	0.070	[-0.230, 0.020]	1.871
**Irony type×WMC**	**-0.120**	**0.043**	**-2.786**	0.005**	**[-0.200**,** -0.040]**	**0.170**
	Simple effects analysis					
High WMC: criticism vs. praise	-0.52	0.064	-0.815	0.418	[ -0.083, 0.179 ]	
**Low WMC: criticism vs. praise**	**-0.172**	**0.090**	**-1.792**	0.009**	**[ 0.036**,** 0.297 ]**	
	Rereading time					
WMC(high vs. low)	0.182	0.170	1.073	0.288	[-0.150, 0.480]	1.844
Irony type (criticism vs. praise)	-0.088	0.084	-1.039	0.305	[-0.250, 0.080]	3.324
**Irony type×WMC**	**-0.148**	**0.063**	**-2.355**	0.019*	**[-0.270**,** -0.020]**	**0.522**
	Simple effects analysis					
High WMC: criticism vs. praise	-0.014	0.090	-0.154	0.878	[ -0.159, 0.197 ]	
Low WMC: criticism vs. praise	-0.161	0.090	-1.792	0.078	**[0.118**,** 0.349 ]**	
	Total reading time					
WMC(high vs. low)	0.064	0.100	0.639	0.526	[-0.150, 0.260]	4.352
**Irony type (criticism vs. praise)**	**-0.189**	**0.056**	**-3.398**	0.001**	**[-0.290**,** -0.070]**	**0.075**
**Irony type×WMC**	**-0.099**	**0.037**	**-2.667**	0.008**	**[-0.170**,** -0.030]**	**0.307**
	Simple effects analysis					
**High WMC: criticism vs. praise**	**-0.140**	**0.059**	**-2.387**	0.020*	**[ 0.016**,** 0.249 ]**	
**Low WMC: criticism vs. praise**	**-0.238**	**0.059**	**-4.059**	< 0.001***	**[ 0.118**,** 0.349 ]**	

Bold font indicates significant results. * = *p* < .05, ** = *p* < .01, *** = *p* < .001. WMC = working memory capacity. For Bayesian analysis, *BF*_01_ < 1 indicates evidence for the alternative hypothesis (H_1_); *BF*_01_ > 1 indicates evidence for the null hypothesis (H_0_)

**Fig. 6:**
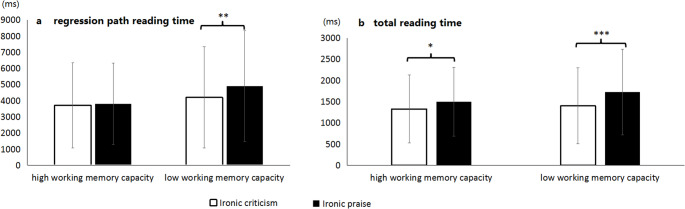
The interaction in regression path reading time (Panel a) and total reading time (Panel b) in the target sentence as a function of irony type and working memory capacity in Experiment 3. The unit of vertical axis is millisecond and the error bars denote standard deviations. * = *p* < .05, ** = *p* < .01, *** = *p* < .001

In the target sentence region, there was a significant main effect of irony type in first-pass reading time (*t* = 2.63, *p* < .05) and total reading time (*t* = 3.398, *p* = .001). A Bayesian analysis converged with these results, supporting an irony-type effect in first-pass reading time (*BF*_01_ = 0.66; HDI [-0.14, -0.02]) and in total reading time (*BF*_01_ = 0.08; HDI [-0.29, -0.07]). Participants took significantly longer to read the target sentence in the ironic praise (first-pass reading time: 634 ± 442ms; total reading time: 1612 ± 907ms) than in the ironic criticism condition (first-pass reading time: 563 ± 369ms; total reading time: 1363 ± 846ms). There was also a significant interaction between irony type and WMC in regression path reading time (*t* = -2.79, *p* < .01), rereading time (*t* = -2.36, *p* < .05) and total reading time (*t* = -3.40, *p* < .01). For the regression path reading time, the simple effect analysis showed that for participants with low WMC, the difference between ironic praise (4905 ± 3439ms) and ironic criticism (4196 ± 3134ms) was significant (*t* = -1.79, *p* < .01; see Fig. 6a). However, for participants with high WMC, there was no significant difference in regression path reading time between ironic praise (3810 ± 2519ms) and ironic criticism (3699 ± 2639ms) (*t* = − 0.82, *p* > .05). For the rereading time, the simple effect analysis showed that for participants with low WMC, the difference between ironic praise (4299 ± 3581ms) and ironic criticism (3660 ± 3255ms) was marginally significant (*t* = -1.79, *p* = .078). For participants with high WMC, there was no significant difference in rereading time between ironic praise (3182 ± 2635ms) and ironic criticism (3152 ± 2711ms) (*t* = − 0.15, *p* = .878). For the total reading time, the simple effect analysis showed that for both low and high WMC participants, the total reading time for ironic praise was significantly longer than that for ironic criticism (high WMC: *t* = -2.39, *p* < .05; low WMC: *t* = -4.06, *p* < .001; see Fig. 6b). However, low WMC participants showed a larger difference in total reading time between ironic praise and ironic criticism (ironic praise: 1728ms, ironic criticism: 1399ms) compared to high WMC participants (ironic praise: 1495ms, ironic criticism: 1326ms), indicating that although both groups had difficulty in reading ironic praise relative to ironic criticism, participants with low WMC were confronted with more difficulties. The Bayesian model comparison likewise favored an interaction (Regression path reading time: *BF*_01_ = 0.17, HDI [-0.20, -0.04]; Reading time: *BF*01 = 0.52, HDI [-0.27, -0.02]; Total reading time: *BF*_01_ = 0.31, HDI [-0.17, -0.03]). Simple-effects contrasts showed that low-WMC readers spent longer on ironic praise than on ironic criticism (Regression path reading time: Δlog = 0.17, HDI [ 0.0, 0.30]; Total reading time: Δlog = 0.23, HDI [0.12, 0.35]). For high-WMC readers, the simple effect was evident only in Total reading time (Δlog = 0.13, HDI [0.02, 0.25]). For Reading time, the low-WMC simple effect was marginal significant in the Bayesian analysis (Δlog = 0.16, HDI [0.12, 0.35]), in line with the borderline *p* value in the LMM analysis.

**Fig. 7:**
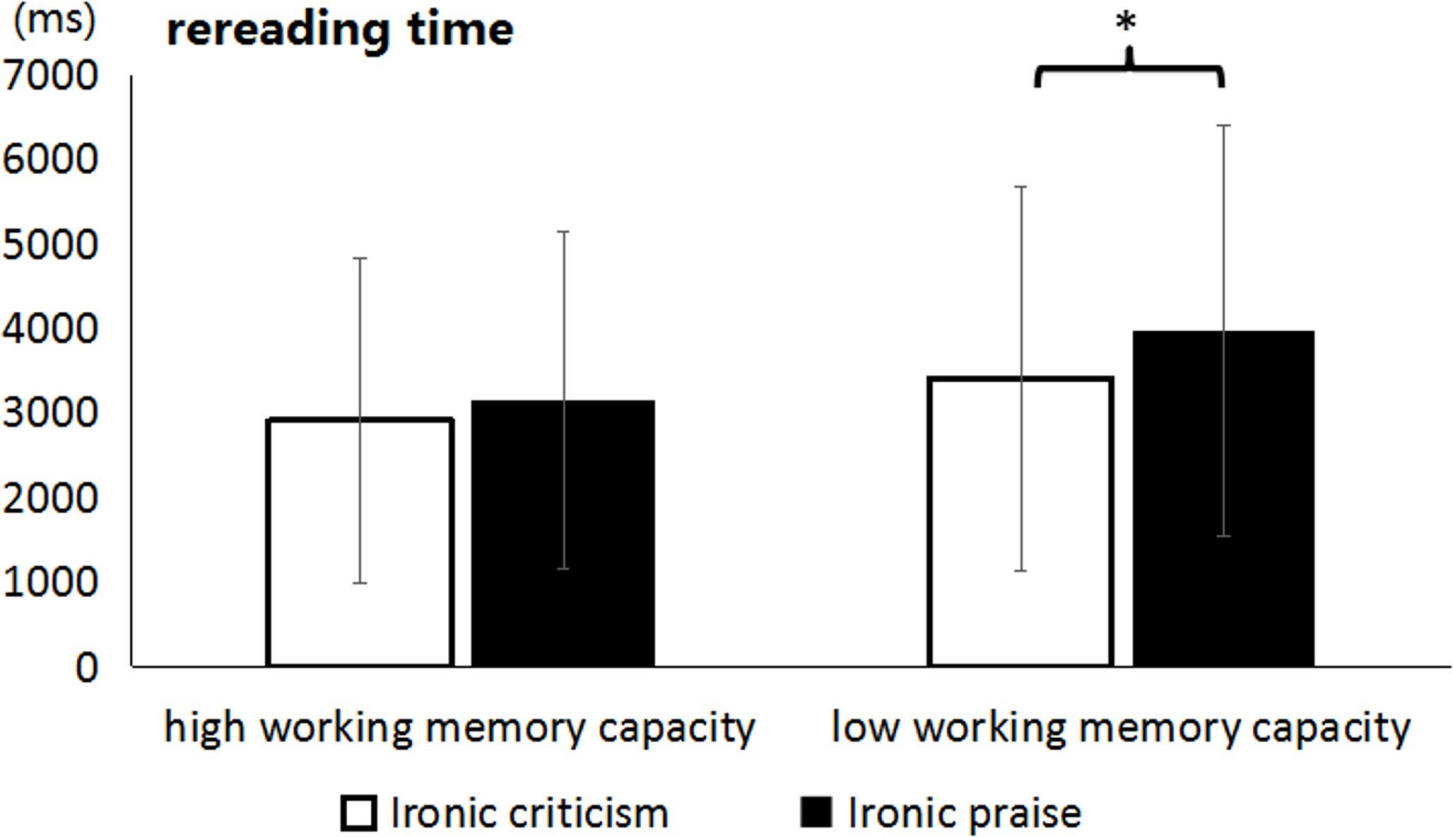
Rereading time for the context region as a function of ironic type and working memory capacity in Experiment 3. The unit of vertical axis is millisecond and the error bars denote standard deviations. Note: * = *p* < .05

The LMM and Bayesian analysis results for the context region are shown in Table 12. For the context region, a significant main effect of WMC emerged in regression path time (*t* = 2.46, *p* < .05), and total reading time (*t* = 2.18, *p* < .05). Bayesian analysis supported these findings with Bayes factors indicating strong evidence for the main effect of WMC in regression path time (*BF*_01_ = 0.41) and total reading time (*BF*_01_ = 0.66). Participants with low WMC had significantly longer regression path reading time and total reading time in the context region than those with high WMC. There was also a significant interaction between irony type and WMC in rereading time (*t* = -2.23, *p* = .03) that closely failed to reach significance in regression path reading time. Simple effect analysis showed that for participants with low WMC, the rereading time for ironic praise (3936 ± 2431ms) was significantly longer than that for ironic criticism (3409 ± 2274ms) (*t* = -2.57, *p* < .05; see Fig. 7). However, for participants with high WMC, there was no significant difference in rereading time between ironic criticism (2908 ± 1930ms) and ironic praise (3142 ± 1994ms) (*t* = -1.23, *p* > .05). Bayesian analysis of the interaction revealed that the contrast between ironic praise and ironic criticism was credible for low WMC participants (Δlog = 0.17, HDI = [0.03, 0.29]).

**Table 12:** The results of the linear mixed-effects model analysis and bayesian analysis for the eye-tracking measures in the context region in experiment 3

	b	SE	t	*p*	95% HDI	BF₀₁
	First-pass reading time					
WMC(high vs. low)	0.102	0.083	1.222	0.227	[-0.060, 0.270]	2.856
Irony type (criticism vs. praise)	-0.088	0.077	-1.154	0.254	[-0.050, 0.100]	9.847
Irony type×WMC	-0.028	0.0479	-0.573	0.566	[-0.060, 0.080]	12.832
	Regression path reading time					
**WMC(high vs. low)**	**0.165**	**0.067**	**2.464**	0.017*	**[0.030**,** 0.300]**	**0.415**
Irony type (criticism vs. praise)	-0.046	0.045	-0.137	0.305	[-0.140, 0.040]	6.104
Irony type×WMC	-0.045	0.024	-1.921	0.055	[-0.100, 0.010]	1.819
	Rereading time					
WMC(high vs. low)	0.189	0.097	1.946	0.057	[-0.020, 0.370]	0.859
Irony type (criticism vs. praise)	-0.124	0.062	-1.993	0.052	[-0.250, 0.000]	1.231
**Irony type×WMC**	**-0.088**	**0.040**	**-2.231**	0.026*	**[-0.160**,** -0.010]**	**0.841**
	Simple effects analysis					
High WMC: criticism vs. praise	-0.080	0.065	-1.227	0.225	[− 0.051, 0.215]	
**Low WMC: criticism vs. praise**	**-0.169**	**0.066**	**-2.571**	0.013*	**[0.029**,** 0.294]**	
	Total reading time					
**WMC(high vs. low)**	**0.163**	**0.075**	**2.183**	0.033*	**[0.000**,** 0.310]**	**0.661**
Irony type (criticism vs. praise)	0.009	0.046	0.204	0.839	[-0.080, 0.100]	10.939
Irony type×WMC	-0.020	0.028	-0.711	0.477	[-0.080, 0.030]	11.721

Bold font indicates significant results

* = *p* < .05, ** = *p* < .01, *** = *p* < .001. WMC= working memory capacity. For Bayesian analysis, *BF*_01_ < 1 indicates evidence for the alternative hypothesis (H₁); *BF*_01_ > 1 indicates evidence for the null hypothesis (H_0_).

The results of LMM and Bayesian analysis for the spillover region are presented in Table 13. The analysis revealed a significant interaction between irony type and working memory capacity in regression path duration (*t* = -2.56, *p* < .05). Bayesian analysis provided robust evidence for this interaction (*BF*_01_= 0.23, HDI [-0.29, -0.04]). Simple effect analysis indicated that participants with low working memory capacity exhibited significantly longer regression path durations when reading ironic praise (2615 ± 2147 ms) compared to ironic criticism (2093 ± 1850 ms) (*t* = 2.33, *p* < .05; Bayesian contrast: Δlog = 0.18, HDI [0.01, 0.34]), whereas no significant difference was observed between ironic praise (2001 ± 1643 ms) and ironic criticism (1970 ± 1681 ms) among participants with high working memory capacity (*t* = -0.30, *p* > .05).

A significant interaction was also found for rereading time (*t* = -2.55, *p* < .05). Bayesian analysis corroborated this interaction (*BF*_01_ = 0.29, HDI [–0.34, − 0.04]). Simple effect analysis demonstrated that participants with low working memory capacity spent significantly more time rereading ironic praise (2486 ± 2180 ms) than ironic criticism (1968 ± 1824 ms) (*t* = -2.09, *p* < .05; Bayesian contrast: Δlog = 0.20, HDI [0.01, 0.40]). Again, no significant difference emerged between ironic praise (1839 ± 1597 ms) and ironic criticism (1818 ± 1647 ms) for participants with high working memory capacity (*t* = -0.08, *p* > .05; Bayesian contrast: Δlog = 0.01, HDI [–0.18, 0.21]).

Finally, a significant interaction emerged for total reading time (*t* = -2.37, *p* < .05; Bayesian analysis: *BF*_01_ = 0.93, HDI [–0.18, − 0.01]). Simple effect analysis revealed a marginally significant difference in total reading time between ironic praise (299 ± 171 ms) and ironic criticism (269 ± 155 ms) among participants with low working memory capacity (*t* = 1.82, *p* = .07; Bayesian contrast: Δlog = 0.10, HDI [–0.01, 0.20]), whereas no significant difference was found between ironic praise (269 ± 157 ms) and ironic criticism (280 ± 174 ms) for participants with high working memory capacity (t = 0.05, *p* = .96; Bayesian contrast: Δlog = 0.002, HDI [–0.11, 0.12]).

**Table 13:** The results of the linear mixed-effects model analysis and bayesian analysis for the eye-tracking measures in the spillover region in experiment 3

	b		SE	t		*p*		95% HDI	BF₀₁
	First-pass reading time								
WMC(high vs. low)	0.023		0.042	0.550		0.584		[-0.060, 0.110]	9.221
Irony type (criticism vs. praise)	-0.018		0.034	-0.545		0.589		[-0.090, 0.050]	11.350
Irony type×WMC	-0.011		0.031	-0.351		0.726		[-0.060, 0.060]	15.892
	Regression path reading time								
WMC(high vs. low)	-0.119		0.083	-1.421		0.162		[-0.050, 0.400]	1.244
Irony type (criticism vs. praise)	0.175		0.122	1.429		0.159		[-0.250, 0.060]	2.753
Irony type×WMC	-0.182		0.071	-2.563		0.010*		[-0.290, -0.040]	0.228
	Simple effects analysis								
High WMC: criticism vs. praise	-0.028		0.092	-0.301		0.764		[-0.154, 0.183]	
**Low WMC: criticism vs. praise**	**-0.210**		**0.090**	**2.332**		0.023*		**[0.012**,** 0.344]**	
	Rereading time								
WMC(high vs. low)	0.206		0.135	1.520		0.134		[-0.080, 0.460]	1.332
Irony type (criticism vs. praise)	-0.105		0.090	-1.167		0.249		[-0.290, 0.070]	2.522
**Irony type×WMC**	**-0.195**		**0.077**	**-2.546**		0.011*		**[-0.340**,** -0.040]**	**0.289**
	Simple effects analysis								
High WMC: criticism vs. praise	-0.008		0.099	-0.079		0.937		[-0.182, 0.210]	
**Low WMC: criticism vs. praise**	**-0.203**		**0.097**	**-2.087**		0.041*		**[0.014**,** 0.397]**	
	Total reading time								
WMC(high vs. low)	0.076		0.065	1.176		0.245		[-0.050, 0.210]	3.673
Irony type (criticism vs. praise)	-0.047		0.050	-0.951		0.347		[-0.150, 0.050]	5.763
**Irony type×WMC**	**-0.100**		**0.042**	**-2.370**		0.018*		**[-0.180**,** -0.010]**	**0.930**
	Simple effects analysis								
High WMC: criticism vs. praise	0.003		0.054	0.050		0.960		[-0.106, 0.115]	
Low WMC: criticism vs. praise	-0.097		0.053	1.820		0.074		[-0.010, 0.204]	

Bold font indicates significant results

* = *p* < .05, ** = *p* < .01, *** = *p* < .001. WMC= working memory capacity. For Bayesian analysis, *BF*_01_ < 1 indicates evidence for the alternative hypothesis (H₁); *BF*_01_ > 1 indicates evidence for the null hypothesis (H_0_)

#### Accuracy in responding to memory and inference questions as a function of irony type and working memory capacity

A 2 (Irony type: ironic criticism vs. ironic praise) ×2 (Question type: text memory vs. text inference) × 2 (WMC: high vs. low) mixed design ANOVA was conducted on response accuracy. The descriptive statistics are presented in Table 14. The results showed a significant main effect of irony type (*F* (1, 55) = 23.73, *p* < .001, *η*_*p*_^*2*^ = 0.3), with lower accuracy for ironic praise (0.81 ± 0.12) compared to ironic criticism (0.86 ± 0.08), indicating greater difficulty in understanding ironic praise. There was also a significant main effect of question type (*F* (1, 55) = 47.72, *p* < .001, *η*_*p*_^*2*^ = 0.47), with higher accuracy for text memory questions (0.86 ± 0.06) than for text inference questions (0.79 ± 0.14), indicating greater difficulty in responding to text inference than text memory questions.

**Table 14:** Accuracy (M ± SD) for memory and inference questions across the different conditions in experiment 3

	High working memory capacity		Low working memory capacity	
	Ironic criticism	Ironic praise	Ironic criticism	Ironic praise
Memory	0.92 (0.06)	0.86 (0.07)	0.90 (0.05)	0.85 (0.07)
Inference	0.83 (0.09)	0.80 (0.17)	0.80 (0.13)	0.73 (0.16)

### Discussion

In Experiment 3, we examined the role of working memory capacity (WMC) in processing ironic criticism versus ironic praise under weak contextual inconsistency. Replicating the pattern from Experiment 1, ironic praise elicited longer first-pass and total reading times than ironic criticism, confirming its greater difficulty when contextual support is limited. More importantly, WMC moderated irony processing: low-WMC readers showed longer regression-path reading times on the target sentence and engaged in more rereading of the prior context for ironic praise than high-WMC readers, suggesting greater reliance on contextual reinspection to resolve the double negation inherent in ironic praise. The total reading time difference between ironic praise and criticism was also larger for low-WMC readers, indicating fewer available cognitive resources for processing demanding ironic forms.

These findings extend prior work in showing that high-WMC readers resolve irony earlier than low-WMC readers (Kaakinen et al., 2014; Olkoniemi et al., 2016, 2019). While previous studies compared ironic to literal statements, we compared two types of irony and found that WMC effects emerged primarily in late integration measures (regression path time, rereading time, total reading time) rather than in early first-pass reading. This supports the view that WMC modulates the timeline of irony processing, particularly during resource-intensive reinterpretation stages (Just & Carpenter, 1992; Yan et al., 2013).

Additionally, a main effect of WMC was observed in the context region, with low-WMC readers exhibiting longer overall processing times, consistent with broader cognitive advantages associated with higher WMC (Du et al., 2022; Fernández et al., 2021; Ye et al., 2018). In the spillover region, an interaction between irony type and WMC further indicated that low-WMC readers experienced prolonged processing for ironic praise, aligning with findings that ironic praise imposes greater cognitive demand (Filik et al., 2016) and that high-WMC facilitates faster irony resolution (Regel et al., 2011).

Taken together, the results underscore that ironic praise is more cognitively taxing than ironic criticism, and that working memory capacity moderates this difficulty mainly during late-stage contextual integration.

## General discussion

In the present study, the eye-tracking method was employed to investigate the processing and comprehension of written irony in Chinese. A key novelty of the present study lies in its examination of irony processing in an Eastern culture using a logographic script, whereas the majority of prior research has been conducted in Western cultures with alphabetic writing systems. By doing so, we were able to discern the extent to which prior research on written irony comprehension generalizes across cultures and scripts. It is not self-evident that cognitive processes in reading, including irony comprehension, generalize across cultures and scripts (Li et al., 2022; Nisbett et al., 2001).

We examined how the context and working memory capacity affect the comprehension of irony. Firstly, it was revealed that no significant difference existed between ironic and literal language comprehension. However, compared to ironic criticism, ironic praise elicited longer reading times in both the context and target sentence regions. Furthermore, it was revealed that when the inconsistency between the prior context and the ironic utterance was strong, there was no difference in reading ironic praise versus ironic criticism. On the other hand, when the inconsistency was weak (lending less support for the ironic interpretation), ironic praise required more processing time than ironic criticism. Our results replicate and extend Western findings (e.g., Pexman & Glenwright, 2007; Rivière et al., 2018) to Chinese, demonstrating that ironic praise is universally more demanding. Notably, the observed delays in first-pass reading mirror Kaakinen et al.’s (2014) findings, suggesting that early detection of pragmatic incongruity transcends script differences. Additionally, individual differences in working memory capacity modulated the processing of ironic praise and ironic criticism under the weak context condition. Participants with low working memory capacity showed a larger difference in total reading time between ironic praise and ironic criticism. Moreover, low-capacity readers required more help from the prior context in reading ironic praise than ironic criticism. This became apparent in regression-path reading time of the target phrases and in rereading time of the context region.

### The effect of context on reading ironic praise and ironic criticism

In Experiment 1, we first examined the influence of evaluation type (praise vs. criticism) on the comprehension of both literal and ironic language in Chinese. The results indicated no main effect of expression type, suggesting that there was no significant difference between the processing of ironic and literal language. This finding contradicts the predictions of the Standard Pragmatic Model. Instead, the results align more closely with the Direct Access View (Gibbs, 1994), indicating that irony does not necessarily incur extra processing cost when supportive context is available. However, we also observed that ironic praise required more processing time than ironic criticism, especially under weak contextual support in Experiment 2. More specifically, under weak inconsistency, ironic praise required significantly longer reading times than ironic criticism in both early (first-pass) and late (regression path, rereading, total time) measures, indicating greater comprehension difficulty when contextual support was lacking. This asymmetry is better explained by frequency-based expectation effects (Regel et al., 2010) and the Pretense Theory (Clark & Gerrig, 1984), which emphasize that ironic praise is less conventional and requires stronger contextual cues for comprehension.

The present results align with and extend prior findings on the dynamics of irony processing. The longer first-pass reading times for ironic praise under weak inconsistency mirror a pattern observed by Kaakinen et al. (2014), who found early pragmatic incongruity detection in an alphabetic script. Notably, our regression path and rereading time effects in Chinese parallel Olkoniemi et al.‘s (2016) demonstration of late-stage integration difficulties for sarcasm in Finnish, suggesting script-independent reliance on contextual reprocessing when irony is ambiguous. Cross-cultural consistency is further highlighted by Filik et al. (2014), whose electrophysiological data (N400/P600) in English corresponded to our observed timeline—early contextual mismatch detection (first-pass effects) followed by late reinterpretation (rereading).

The absence of irony type differences under strong contextual inconsistency replicates Rivière et al.‘s (2018) findings in French, supporting the universality of contextual salience in resolving pragmatic conflicts (Colston & O’Brien, 2000; Colston, 2002; Gerrig & Goldvarg, 2000; Hancock et al., 2000; Huang & Wang, 2013; Ivanko & Pexman, 2003; Pexman & Zvaigzne, 2004; Pexman & Glenwright, 2007; Rivière et al., 2018; Zhang & Zhang, 2006). Thus, our results strongly support the view that context is a powerful linguistic asset in irony reading. Strong context inconsistency means that there is a clear discrepancy in meaning between the information provided by the prior context and the information conveyed in the target sentence. The discrepancy can accelerate the reader’s speed in comprehending the ironic meaning inherent in the target utterance (Gibbs, 1994; Hancock et al., 2000; Rivière et al., 2018; Utsumi, 2000). These results are consistent with the Pretense Theory (Clark & Gerrig, 1984), which states that the literal meaning of ironic praise is negative in its tone and contains less presumptions towards positive expectation people implicitly hold. Therefore, the comprehension of ironic praise requires more explicit prior context information (Hancock et al., 2000), and if the prior context information is not clear enough, the comprehension of ironic praise will be significantly impeded (Gibbs, 1994; Utsumi, 2000). Lack of contextual support coupled with the extra processing cost inherent in comprehending ironic praise leads to ironic praise being more difficult to read than ironic criticism.

A key issue in irony processing is related to the confound of emotional valence, which reflects the inherent asymmetry between negative and positive stimuli. According to the tinge hypothesis (Dews et al., 1995), irony attenuates the emotional intensity of literal statements: ironic criticism softens strong negativity, while ironic praise subtly undercuts positivity, reflecting a negativity bias in social communication (Rockwell, 2007). Affective priming studies show that negative stimuli are processed more intensely and remembered longer than positive ones (Ito et al., 1998). The observed longer total reading times for ironic praise likely result from resolving pragmatic incongruity and processing its emotionally charged intent (Colston, 1997). The delayed late-stage processing may be due to the counter-normative nature of ironic praise, which demands greater cognitive effort to interpret its implied negativity (Pexman & Olineck, 2002). These results support a dual-process framework, where early irony detection depends on contextual incongruity, and late processing is influenced by valence, aligning with the tinge hypothesis’ emphasis on emotional attenuation (Dews et al., 1995). This perspective explains how pragmatic and emotional factors jointly shape irony comprehension.

### The effect of working memory capacity on the processing of ironic praise and ironic criticism

Effects of working memory capacity on the processing of ironic praise and ironic criticism under weak contextual inconsistency was examined in Experiment 3. Participants with low working memory capacity spent significantly longer regression path time and rereading time when reading utterances expressing ironic praise than ironic criticism, while participants with high working memory capacity did not show any significant differences reading the two types of irony. In total reading time, both high and low working memory capacity participants showed significantly longer processing time for ironic praise than ironic criticism, but the difference was larger for participants with low working memory capacity. For the prior context region, the rereading time for ironic praise was significantly longer than for ironic criticism among participants with low working memory capacity, but not among participants with high working memory capacity.

The WMC effects observed here refine existing results on individual differences in irony processing. Kaakinen et al. (2014) showed that high-WMC readers resolve irony earlier in first-pass reading, whereas low-span readers do it with a delay, as indexed by increased look-backs to ironic versus literal statements (Kaakinen et al., 2014; Olkoniemi et al., 2016). Our Chinese participants exhibited WMC modulation primarily in late measures (regression path/rereading). While this may partially reflect greater demands in decoding a logographic script (Li et al., 2022), it could also indicate strategic preferences or reading adaptations shaped by contextual and syntactic structures, particularly under conditions of weak contextual support. This aligns with Olkoniemi et al.‘s (2016) proposal that high-WMC advantages emerge most strongly during resource-intensive stages—here, the double negation required for ironic praise (Giora, 1995).

Another explanation is based on the attentional resource view (Just & Carpenter, 1992). As the comprehension of ironic praise contains double negation (a negatively toned utterance needs to be negated; Giora, 1995), it is more difficult to understand than the single negation (a positively toned utterance is negated) required for understanding ironic criticism. Therefore, understanding ironic praise requires more attentional resources. In Experiment 3, the weak context did not clearly signal an ironic interpretation, increasing the processing difficulty for ironic praise. Yet, participants with high working memory capacity had more assignable attentional resources to readily compute the intended meaning of the text. Although high-capacity readers spent more time reading utterances expressing ironic praise than on those expressing ironic criticism, this difference was smaller compared to that of low-capacity readers. Moreover, they did not need to devote extra time to the context region of the ironic praise than that of the ironic criticism. This suggests that they were able to retain in mind key contextual elements relevant in understanding ironic praise. On the other hand, this was not the case with the low-capacity readers, who returned to the context area of the ironic praise for additional reprocessing.

The effects of working memory capacity on the cognitive processing of ironic praise and ironic criticism mainly manifested at the late integration stage of text reading. The eye-tracking data for the context area, the target sentence and the spillover region supported this conclusion. At this stage, the effective utilization of various cues, the maintenance of multiple potential interpretations in the mind, and the effective extraction and analysis of context and target sentence information played an important role in the eventual understanding of the irony consistent with the context. In all, the present study demonstrates that working memory capacity is an important individual factor functioning in the late integration stage of irony reading.

There was also evidence suggesting that working memory capacity is related to the reading comprehension ability in general (Daneman & Merikle, 1996). The high-capacity readers spent generally less time reading the context region than the low-capacity readers, irrespective of the type of irony. This was true both for first-pass and second-pass reading. In other words, participants with high working memory capacity may have a better ability than those with low working memory capacity in processing syntactic, semantic and pragmatic information required for successful comprehension. Therefore, the differences between high and low working memory capacity participants in reading ironic praise and ironic criticism texts might also reflect differences in their reading abilities. This issue is worth exploring in further research.

While previous research on irony comprehension has focused primarily on alphabetic languages, this study investigates how the logographic nature of Chinese shapes its processing. The results provide valuable insights into the debate between universal and language-specific models of irony processing. Firstly, despite script differences, we find cross-cultural parallels in irony comprehension: ironic praise proves more challenging than ironic criticism, which replicates Western findings (Filik et al., 2014; Olkoniemi et al., 2016). Secondly, the visual complexity and limited grapheme-phoneme correspondence of Chinese logographic script may impose distinct working memory demands, potentially delaying initial lexical access (Li et al., 2022). Yet, such script-level processing differences likely represent only one contributing factor to cross-cultural variation in irony comprehension. Irony understanding fundamentally relies on pragmatic inference, contextual integration, and shared cultural schemas. Thus, observed differences between Chinese and Western readers may stem more substantially from divergent cultural scripts, pragmatic norms, and irony usage patterns. For example, Western irony often employs exaggeration or marked prosody, whereas Chinese irony tends to be contextually embedded and indirect. The heavy reliance on visual-orthographic analysis in Chinese may slow literal meaning retrieval, while subsequent integration of cultural and pragmatic cues shapes later-stage processing patterns—including eye-movement indices during extended reading phases.

## Limitations and future research

One limitation is that the role of emotional valence was not considered in the present study. We fully acknowledge that our initial interpretation overlooked established theories such as the tinge hypothesis and affective asymmetry, which are indeed critical to understanding irony comprehension. While our design did not explicitly measure valence, we acknowledge this limitation: subsequent investigations may benefit from systematically incorporating emotional valence as a variable in irony processing studies.

Another limitation is the rather small number of observations in each condition. According to previous research (Brysbaert & Stevens, 2018), there should be at least 1600 observations (number of subjects * number of items) per condition to ensure enough power for LMM (linear mixed-effects modelling). While our observations per condition (360 in Experiment 1, 672 in Experiment 2) were below 1,600 and although the Bayesian mixed-effects modeling verified that the statistical power was sufficient, future studies should increase the number of data points to improve the analysis power.

The third limitation of this study stems from the material settings. To ensure pragmatic naturalness, the prior contexts were not fully identical between the different versions. Although this enhances ecological validity, it might also introduce the possibility that differences in contextual factors (e.g., inferential demand) may have contributed to the observed effects. Future research should aim to replicate these findings using identically controlled contexts by manipulating only a critical element within a constant narrative frame. This would allow for a more precise attribution of the effects to irony processing mechanisms.

Future research should also consider incorporating a more diverse set of experimental and participant variables to further refine the understanding of irony processing. Specifically, contextual surprisal should be quantified to assess its impact on early fixation measures. Individual differences in reading speed should be examined as a covariate for late processing measures. Additionally, comparing monolingual and bilingual participants could clarify whether the observed effects relate to domain-general executive resources or language-specific pragmatic inferences. Investigating these factors would help disentangle the contributions of linguistic expectation, processing capacity, and cognitive reserve to irony comprehension.

## Conclusion

Based on the three experiments, the following conclusions can be drawn: (1) Irony processing is not necessarily more cognitively demanding than literal language when a supportive context is provided, though ironic praise requires greater contextual integration than ironic criticism.

(2) Context is a powerful linguistic cue in irony reading; a strong discrepancy in meaning between the prior context and the ironic text helps comprehend ironic praise in reading. The context effect on irony is observed in the first-pass reading and spills over to the later processing stage. (3) Working memory capacity modulates the online comprehension of written irony under the weak context inconsistency condition. Readers with high working memory capacity comprehend ironic praise more readily than readers with low working memory capacity. The modulation by individual differences in working memory capacity manifests at the late integration stage of irony reading. (4) The effects were obtained among Chinese participants reading a logographic script. This means that the greater difficulty in reading ironic praise and the modulation of working memory capacity previously observed in alphabetic languages can generalize across cultures and scripts.

## Supplementary Information

Below is the link to the electronic supplementary material.

Supplementary Material 1 (DOCX 53.6 KB)

## Data Availability

Data and materials are openly available on the Open Science Framework at https://osf.io/a3mkq/. This study was preregistered in OSF before conducting the research.

## Code Availability

R Code is openly available at https://osf.io/a3mkq/files/osfstorage.
