# Supplementary material for: Context and working memory capacity affect the processing of written irony in chinese: an eye-tracking study
Source: Psychol Res. 2026 Mar 31;90(2):67. doi: 10.1007/s00426-026-02258-w (PMC13038724; doi:10.1007/s00426-026-02258-w)
Supplement: Supplementary file 1 — Supplementary Material 1 (DOCX 53.6 KB) [file 426_2026_2258_MOESM1_ESM.docx]

***Supplementary tables:***

**Table 1** *Examples of Experimental Materials for Each Condition in Experiment 1 (In Chinese)*

|  | Criticism | | Praise | |
| --- | --- | --- | --- | --- |
|  | Irony | Literal | Irony | Literal |
| Context | 一大早，你和王伟乘公交车去火车站，不料公交车出了故障，你们等了好久才等来另一辆公交车，好不容易到了火车站，但已错过了火车。 | 一大早，你和王伟乘公交车去火车站，不料公交车出了故障，你们等了好久才等来另一辆公交车，好不容易到了火车站，但已错过了火车。 | 你平时很节俭，总是不舍得花钱买贵重的东西，但你的好朋友过生日时，你花费了很多钱给你朋友买了一个名牌包包。 | 你平时很节俭，总是不舍得花钱买贵重的东西，但你的好朋友过生日时，你花费了很多钱给你朋友买了一个名牌包包。 |
| Target sentence | “我们今天可真幸运啊！”王伟说。 | “我们今天可真倒霉啊！”王伟说。 | “你这人确实很抠门啊！”朋友说。 | “你这人其实很大方啊！”朋友说。 |
|  |  |  |  |  |
| Ending sentence | 你们去人工售票台改签火车票。 | 你们去人工售票台改签火车票。 | 朋友给了你一个大大的拥抱。 | 朋友给了你一个大大的拥抱。 |
| Memory question | 你们没赶上火车。 | 你们没赶上火车。 | 你是个节俭的人。 | 你是个节俭的人。 |
| Inference question | 王伟觉得你们今天不幸运。 | 王伟觉得你们今天不幸运。 | 朋友觉得你对她很大方。 | 朋友觉得你对她很大方。 |

**Table 2 *Results of the statistical power test for the degree of irony in the context region of Experiment 1 within the LMM model***

| *Eye-tracking measures* | *b* | *SE* | *t (df)* | *p* |
| --- | --- | --- | --- | --- |
| FPRT | -0.015 | 0.016 | -0.960(702.3) | 0.338 |
| RPRT | -0.003 | 0.013 | -0.262(640.9) | 0.794 |
| TRT | <0.001 | 0.015 | 0.007(696.2) | 0.994 |
| RRT | 0.017 | 0.029 | 0.587(861.2) | 0.557 |

Note:

FPRT: First-pass reading time; RPRT: Regression path reading time; TRT: Total reading time; RRT: Rereading time

**Table 3 *Results of the statistical power test for the degree of irony in the target region of Experiment 1 within the LMM model***

| *Eye-tracking measures* | *b* | *SE* | *t (df)* | *p* |
| --- | --- | --- | --- | --- |
| FPRT | -0.003 | 0.023 | -0.124(895.9) | 0.901 |
| RPRT | 0.001 | 0.023 | 0.058(688.5) | 0.954 |
| TRT | 0.019 | 0.021 | 0.909(701.8) | 0.363 |
| RRT | -0.010 | 0.046 | -0.21(807) | 0.834 |

Note:

FPRT: First-pass reading time; RPRT: Regression path reading time; TRT: Total reading time; RRT: Rereading time

**Table 4 *Results of the statistical power test for the degree of irony in the spillover region of Experiment 1 within the LMM model***

| *Eye-tracking measures* | *b* | *SE* | *t (df)* | *p* |
| --- | --- | --- | --- | --- |
| FPRT | 0.002 | 0.017 | 0.087(625.7) | 0.931 |
| RPRT | 0.056 | 0.036 | 1.54(595.2) | 0.124 |
| TRT | -0.004 | 0.024 | -0.169(647.6) | 0.866 |
| RRT | 0.107 | 0.055 | 1.938(671.7) | 0.053 |

Note:

FPRT: First-pass reading time; RPRT: Regression path reading time; TRT: Total reading time; RRT: Rereading time

**Table 5** *The variance inflation factor (VIF) values for all eye movement indices in Experiment 1*

| ROI |  | degree of irony | Expression type | Evaluation type | Expression type × Evaluation type |
| --- | --- | --- | --- | --- | --- |
| **Context region** | First-pass reading time | 3.156 | 3.152 | 1.003 | <1.001 |
|  | Regression path reading time | 2.291 | 2.286 | 1.002 | 1.003 |
|  | Total reading time | 4.043 | 4.038 | 1.003 | 1.001 |
|  | Rereading time | 2.797 | 2.794 | 1.002 | <1.001 |
| **Target sentence region** | First-pass reading time | 6.286 | 6.268 | 1.007 | 1.009 |
|  | Regression path reading time | 2.71 | 2.706 | 1.003 | 1.001 |
|  | Total reading time | 4.319 | 4.315 | 1.003 | 1.001 |
|  | Rereading time | 3.103 | 3.099 | 1.003 | <1.001 |
| **Spillover region** | First-pass reading time | 4.814 | 4.806 | 1.007 | 1.005 |
|  | Regression path reading time | 2.836 | 2.835 | 1.003 | <1.001 |
|  | Total reading time | 4.279 | 4.275 | 1.005 | 1.003 |
|  | Rereading time | 3.246 | 3.244 | 1.004 | <1.001 |

**Table 6** *Examples of Experimental Materials for Each Condition in Experiment 2 (In Chinese)*

|  | Ironic Criticism | | Ironic Praise | |
| --- | --- | --- | --- | --- |
|  | strong context inconsistency | weak context inconsistency | strong context inconsistency | weak context inconsistency |
| Context | 周末，你家里大扫除，妈妈让你打扫自己的房间，你拿鸡毛掸子随便一掸就算完事了，房间里还特别乱、灰尘也特别多。 | 周末，你家里大扫除，妈妈让你打扫自己的房间，你好好打扫了一番，当妈妈去检查时，地板上还零乱的放着几本书。 | 离放暑假还有一个月，你和老乡小华已经开始网上订票，你眼疾手快的抢到一张三折的机票。所有费用加起来比坐火车回家还便宜的多。 | 离放暑假还有一个月，你和老乡小华已经开始网上订票，你眼疾手快的抢到一张七折的机票。所有费用加起来只比坐高铁回家贵一点。 |
| Target sentence | “你打扫得可真干净啊！”妈妈说。 | “你打扫得可真干净啊！”妈妈说。 | “你这张机票可真贵啊！”小华说。 | “你这张机票可真贵啊！”小华说。 |
|  |  |  |  |  |
| Ending sentence | 你准备去把脏衣服也洗一洗。 | 你准备去把脏衣服也洗一洗。 | 小华继续在网上抢回家的票。 | 小华继续在网上抢回家的票。 |
| Memory question | 你和妈妈在一起大扫除 | 你和妈妈在一起大扫除 | 你和朋友小华一起订票。 | 你和朋友小华一起订票。 |
| Inference question | 妈妈对你打扫的房间不满意。 | 妈妈对你打扫的房间不满意。 | 小华很羡慕你能抢到这张机票。 | 小华很羡慕你能抢到这张机票。 |

**Table 7 *Results of the statistical power test for the degree of irony in the context region of Experiment 2 within the LMM model***

| *Eye-tracking measures* | *b* | *SE* | *t (df)* | *p* |
| --- | --- | --- | --- | --- |
| FPRT | 0.063 | 0.097 | -0.649 (88.0) | 0.518 |
| RPRT | -0.1 | 0.111 | -0.908 (88.8) | 0.366 |
| TRT | -0.065 | 0.112 | -0.581 (89.3) | 0.562 |
| RRT | -0.136 | 0.171 | -0.796 (89.5) | 0.428 |

Note:

FPRT: First-pass reading time; RPRT: Regression path reading time; TRT: Total reading time; RRT: Rereading time

**Table 8 *Results of the statistical power test for the degree of irony in the target region of Experiment 2 within the LMM model***

| *Eye-tracking measures* | *b* | *SE* | *t (df)* | *p* |
| --- | --- | --- | --- | --- |
| FPRT | -0.217 | 0.186 | -1.166 (88.2) | 0.137 |
| RPRT | -0.274 | 0.162 | -1.693 (88.6) | 0.094 |
| TRT | -0.197 | 0.138 | -1.427 (88.3) | 0.157 |
| RRT | -0.32 | 0.215 | -1.489 (88.8) | 0.14 |

Note:

FPRT: First-pass reading time; RPRT: Regression path reading time; TRT: Total reading time; RRT: Rereading time

**Table 9 *Results of the statistical power test for the degree of irony in the spillover region of Experiment 2 within the LMM model***

| *Eye-tracking measures* | *b* | *SE* | *t (df)* | *p* |
| --- | --- | --- | --- | --- |
| FPRT | 0.019 | 0.063 | 0.304 (95.1) | 0.762 |
| RPRT | -0.38 | 0.234 | -1.628 (91.8) | 0.107 |
| TRT | 0.001 | 0.119 | 0.004 (90.4) | 0.997 |
| RRT | -0.281 | 0.236 | -1.192 (89.6) | 0.236 |

Note:

FPRT: First-pass reading time; RPRT: Regression path reading time; TRT: Total reading time; RRT: Rereading time

**Table 10** *The variance inflation factor (VIF) values for all eye movement indices in Experiment 2*

| ROI |  | degree of irony | Irony type | Context inconsistency | Irony type × Context inconsistency |
| --- | --- | --- | --- | --- | --- |
| **Context region** | First-pass reading time | 1.443 | 1.089 | 1.331 | 1.021 |
|  | Regression path reading time | 1.455 | 1.092 | 1.341 | 1.020 |
|  | Total reading time | 1.451 | 1.091 | 1.338 | 1.020 |
|  | Rereading time | 1.455 | 1.092 | 1.345 | 1.018 |
| **Target sentence region** | First-pass reading time | 1.456 | 1.345 | 1.089 | 1.016 |
|  | Regression path reading time | 1.466 | 1.350 | 1.093 | 1.016 |
|  | Total reading time | 1.457 | 1.343 | 1.093 | 1.016 |
|  | Rereading time | 1.467 | 1.350 | 1.092 | 1.018 |
| **Spillover region** | First-pass reading time | 1.414 | 1.094 | 1.302 | 1.016 |
|  | Regression path reading time | 1.432 | 1.095 | 1.32 | 1.015 |
|  | Total reading time | 1.421 | 1.096 | 1.307 | 1.016 |
|  | Rereading time | 1.430 | 1.089 | 1.326 | 1.015 |

**Table 11** *Bayesian analysis for the different eye-tracking measures for*

*the context region in Experiment 2*

|  | 95% HDI | *BF_01_* |
| --- | --- | --- |
| First-pass reading time | | |
| Irony type: Criticism vs. Praise | [-0.080, 0.090] | 9.807 |
| Context inconsistency: Strong vs. Weak | [-0.060, 0.130] | 6.562 |
| Irony type × Context inconsistency | [-0.190, 0.140] | 4.894 |
| Regression path reading time | | |
| Irony type: Criticism vs. Praise | [-0.120, 0.030] | 5.003 |
| Context inconsistency: Strong vs Weak | [-0.070, 0.090] | 9.564 |
| Irony type × Context inconsistency | [-0.220, 0.060] | 3.089 |
| Rereading time | | |
| Irony Type: Criticism vs. Praise | [-0.210, 0.000] | 1.082 |
| Context inconsistency: Strong vs Weak | [-0.100, 0.140] | 6.676 |
| Irony type × Context inconsistency | [-0.310, 0.100] | 2.286 |
| Total reading time | | |
| Irony type: Criticism vs. Praise | [-0.100, 0.050] | 8.801 |
| Context inconsistency: Strong vs. Weak | [-0.080, 0.100] | 8.79 |
| Irony type × Context Inconsistency | [-0.230, 0.070] | 3.284 |

Note: For Bayesian analysis, *BF_01_* >1 indicates evidence for the null hypothesis (H0).

The 95%HDI including zero indicates no significant effect .

**Table 12** *Bayesian analysis for the different eye-tracking measures for*

*the spillover region in Experiment 2*

|  | 95% HDI | *BF_01_* |
| --- | --- | --- |
| First-pass reading time | | |
| Irony type: Criticism vs. Praise | [-0.160, 0.030] | 3.651 |
| Context inconsistency: Strong vs. Weak | [-0.070, 0.130] | 6.562 |
| Irony type × Context inconsistency | [-0.260, 0.100] | 2.918 |
| Regression path reading time | | |
| Irony type: Criticism vs. Praise | [-0.100, 0.260] | 3.089 |
| Context inconsistency: Strong vs Weak | [-0.180, 0.230] | 3.331 |
| Irony type × Context inconsistency | [-0.500, 0.190] | 1.498 |
| Rereading time | | |
| Irony Type: Criticism vs. Praise | [-0.090, 0.300] | 1.082 |
| Context inconsistency: Strong vs Weak | [-0.210, 0.240] | 6.676 |
| Irony type × Context inconsistency | [-0.540, 0.240] | 1.410 |
| Total reading time | | |
| Irony type: Criticism vs. Praise | [-0.150, 0.030] | 4.422 |
| Context inconsistency: Strong vs. Weak | [-0.050, 0.150] | 5.491 |
| Irony type × Context Inconsistency | [-0.200, 0.160] | 4.592 |

Note: For Bayesian analysis, *BF_01_* >1 indicates evidence for the null hypothesis (H0).

The 95% HDI including zero indicates no significant effect .

**Table 13 *Results of the statistical power test for the degree of irony in the context region of Experiment 3 within the LMM model***

| *Eye-tracking measures* | *b* | *SE* | *t (df)* | *p* |
| --- | --- | --- | --- | --- |
| FPRT | -0.001 | 0.099 | -0.008 (44.8) | 0.993 |
| RPRT | -0.048 | 0.114 | -0.418 (44.9) | 0.678 |
| TRT | -0.039 | 0.118 | -0.333 (44.8) | 0.741 |
| RRT | -0.090 | 0.161 | -0.558 (44.8) | 0.580 |

Note:

FPRT: First-pass reading time; RPRT: Regression path reading time; TRT: Total reading time; RRT: Rereading time

**Table 14 *Results of the statistical power test for the degree of irony in the target region of Experiment 3 within the LMM model***

| *Eye-tracking measures* | *b* | *SE* | *t (df)* | *p* |
| --- | --- | --- | --- | --- |
| FPRT | -0.017 | 0.08 | -0.218 (44.9) | 0.828 |
| RPRT | -0.086 | 0.156 | -0.551 (44.8) | 0.584 |
| TRT | 0.001 | 0.143 | 0.007 (44.8) | 0.994 |
| RRT | -0.056 | 0.218 | -0.256 (44.9) | 0.8 |

Note:

FPRT: First-pass reading time; RPRT: Regression path reading time; TRT: Total reading time; RRT: Rereading time

**Table 15 *Results of the statistical power test for the degree of irony in the spillover region of Experiment 3 within the LMM model***

| *Eye-tracking measures* | *b* | *SE* | *t* (*df*) | *p* |
| --- | --- | --- | --- | --- |
| FPRT | 0.006 | 0.087 | 0.074 (44.9) | 0.941 |
| RPRT | -0.252 | 0.215 | -1.171 (45.6) | 0.248 |
| TRT | -0.041 | 0.128 | -0.324 (45.0) | 0.747 |
| RRT | -0.41 | 0.233 | -1.760 (45.9) | 0.085 |

Note:

FPRT: First-pass reading time; RPRT: Regression path reading time; TRT: Total reading time; RRT: Rereading time

**Table 16** *The variance inflation factor (VIF) values for all eye movement indices in Experiment 3*

| ROI |  | degree of irony | Irony type | working memory capacity | Irony type × working memory capacity |
| --- | --- | --- | --- | --- | --- |
| **Context region** | First-pass reading time | 1.017 | 1.017 | <1.001 | <1.001 |
|  | Regression path reading time | 1.017 | 1.018 | <1.001 | <1.001 |
|  | Total reading time | 1.017 | 1.017 | <1.001 | <1.001 |
|  | Rereading time | 1.017 | 1.017 | <1.001 | <1.001 |
| **Target sentence region** | First-pass reading time | 1.017 | 1.017 | <1.001 | <1.001 |
|  | Regression path reading time | 1.017 | 1.017 | <1.001 | <1.001 |
|  | Total reading time | 1.017 | 1.017 | <1.001 | <1.001 |
|  | Rereading time | 1.017 | 1.017 | <1.001 | <1.001 |
| **Spillover region** | First-pass reading time | 1.030 | 1.034 | <1.001 | 1.004 |
|  | Regression path reading time | 1.030 | 1.034 | <1.001 | 1.003 |
|  | Total reading time | 1.031 | 1.036 | <1.001 | 1.004 |
|  | Rereading time | 1.035 | 1.038 | <1.001 | 1.003 |

To assess the robustness of our key conclusions to prior distributions, we performed a sensitivity analysis by refitting the models with alternative priors for the slope coefficients: Normal(0, 0.1) and Normal(0, 1). A wider (0, 1) and a narrower (0, 0.1) prior to compare the impact of prior range on posterior results, systematically assessed the robustness of model inferences, and verified whether the conclusions were overly reliant on subjective prior specifications. The Bayes Factors (*BF*₀₁) and 95% Highest Density Intervals (HDI) for all critical effects (e.g., the interactions reported in the results tables) remained stable, confirming that our main findings were robust to the choice of prior. Detailed results of the sensitivity analysis across the three experiments were provided in Supplementary Table 17-19.

**Table 17** *Prior sensitivity analysis for the* *key effects in Experiment 1*

| ROI |  | Normal(0,1) | | Normal(0,0.1) | |
| --- | --- | --- | --- | --- | --- |
|  |  | 95% HDI | *BF_01_* | 95% HDI | *BF_01_* |
| Target sentence region | First-pass reading time | | |  |  |
|  | Expression type × Evaluation type | [-0.030,0.310] | 0.820 | [-0.050, 0.210] | 0.711 |
|  | Regression path reading time | | |  |  |
|  | Expression type × Evaluation type | [0.002,0.290] | 0.778 | [0.000, 0.210] | 0.625 |
|  | Total reading time | | |  |  |
|  | Evaluation type（criticism vs. praise） | [-0.170, -0.040] | 0.177 | [-0.160, -0.030] | 0.033 |
|  | Expression type × Evaluation type | [0.020, 0.250] | 0.434 | [0.020, 0.190] | 0.566 |
| Context region | Rereading time | | |  |  |
|  | Expression type × Evaluation type | [0.009,0.350] | 0.589 | [0.030, 0.230] | 0.493 |

Note: For Bayesian analysis, *BF_01_* >1 indicates evidence for the null hypothesis (H0). The 95% HDI including zero indicates no significant effect .

**Table 18** *Prior sensitivity analysis for the* *key effects in Experiment 2*

| ROI |  | Normal(0,1) | | Normal(0,0.1) | |
| --- | --- | --- | --- | --- | --- |
|  |  | 95% HDI | *BF_01_* | 95% HDI | *BF_01_* |
| Target sentence region | First-pass reading time | | |  |  |
|  | Irony type × Context inconsistency | [-0.370,-0.050] | 0.416 | [-0.250, -0.000] | 0.264 |
|  | Regression path reading time | | |  |  |
|  | Irony type × Context inconsistency | [-0.470,-0.120] | 0.050 | [-0.290, -0.030] | 0.091 |
|  | Rereading time | | |  |  |
|  | Irony type × Context inconsistency | [-0.510,0.110] | 0.092 | [-0.290, -0.010] | 0.161 |
|  | Total reading time | | |  |  |
|  | Irony type: Criticism vs. Praise | [-0.220, -0.040] | 0.276 | [-0.200, -0.040] | 0.042 |

Note: For Bayesian analysis, *BF_01_* >1 indicates evidence for the null hypothesis (H0). The 95% HDI including zero indicates no significant effect .

**Table 19** *Prior sensitivity analysis for the* *key effects in Experiment 3*

| ROI |  | Normal(0,1) | | Normal(0,0.1) | |
| --- | --- | --- | --- | --- | --- |
|  |  | 95% HDI | *BF_01_* | 95% HDI | *BF_01_* |
| Target sentence region | First-pass reading time | | |  |  |
|  | Irony type (criticism vs. praise) | [-0.140,-0.020] | 0.749 | [-0.130, -0.020] | 0.186 |
|  | Regression path reading time | | |  |  |
|  | Irony type×WMC | [-0.200,-0.040] | 0.326 | [-0.180, -0.030] | 0.072 |
|  | Rereading time | | |  |  |
|  | Irony type×WMC | [-0.270, -0.020] | 0.280 | [-0.210, -0.000] | 0.246 |
|  | Total reading time | | |  |  |
|  | Irony type (criticism vs. praise) | [-0.300, -0.070] | 0.137 | [-0.230, -0.040] | 0.049 |
|  | Irony type×WMC | [-0.170, -0.030] | 0.617 | [-0.160, -0.020] | 0.107 |
| Context region | Regression path reading time | | |  |  |
|  | WMC（high vs. low） | [0.030,0.300] | 0.780 | [0.010, 0.230] | 0.230 |
|  | Rereading time | | |  |  |
|  | Irony type×WMC | [-0.250,-0.000] | 0.464 | [-0.150, -0.000] | 0.323 |
|  | Total reading time | | |  |  |
|  | WMC（high vs. low） | [0.002,0.320] | 0.780 | [-0.001, 0.220] | 0.375 |
| Spillover region | Regression path reading time | | |  |  |
|  | Irony type×WMC | [-0.290,-0.050] | 0.442 | [-0.230,-0.020] | 0.145 |
|  | Rereading time | | |  |  |
|  | Irony type×WMC | [-0.340,-0.040] | 0.519 | [-0.240,-0.000] | 0.211 |
|  | Total reading time | | |  |  |
|  | Irony type×WMC | [-0.180,-0.010] | 0.994 | [-0.160,-0.000] | 0.324 |

Note: For Bayesian analysis, *BF_01_* >1 indicates evidence for the null hypothesis (H0). The 95% HDI including zero indicates no significant effect .
